# Critical changes in whole-brain gene networks in response to small-cell lung cancer as revealed by single-nucleus RNA sequencing

**DOI:** 10.3389/fimmu.2026.1860628

**Published:** 2026-06-02

**Authors:** Jingwei Duan, Qi Fu, Yongkun Huo, Caifang Wang, Yuke Shen, Mingtian Zhong, Xiaodong Ma, Ming Liu

**Affiliations:** 1 Key Laboratory of Brain, Cognition and Education Sciences, Ministry of Education, Institute for Brain Research and Rehabilitation, and Guangdong Key Laboratory of Mental Health and Cognitive Science, South China Normal University, Guangzhou, Guangdong, China; 2 National Center for Respiratory Medicine, National Clinical Research Center for Respiratory Disease, State Key Laboratory of Respiratory Disease, Guangzhou Institute of Respiratory Health, The First Affiliated Hospital of Guangzhou Medical University, Guangzhou, China

**Keywords:** endothelial, GABAergic inhibitory neurons, GAD2, lung-brain crosstalk, SCLC, single-nucleus RNA-seq

## Abstract

**Background:**

Small-cell lung cancer (SCLC) is a highly aggressive neuroendocrine malignancy in which neural activity is implicated in tumor progression. Nevertheless, whether primary SCLC elicits systemic effects on the brain remains uncertain.

**Methods:**

Using an *Rb1/Trp53/Myc*-driven SCLC mouse model, we performed whole-brain single-nucleus RNA sequencing (n = 48,686 nuclei) integrated with transcriptomic and metabolomic analyses of lung tumors and plasma, validating our findings across public SCLC cohorts. Pharmacological inhibition of GABA_A_ receptors with flumazenil was also applied *in vivo*.

**Results:**

We observed widespread, cell-type-resolved transcriptional alterations across the brain in tumor-bearing mice. *Gad2* expression broadly increased in basal ganglia cells and GABAergic inhibitory neurons. Concurrently, oligodendrocyte precursor cells exhibited impaired maturation, accompanied by coordinated downregulation of myelination-related genes (*Mbp*, *Plp1*, *Mobp*). Metabolomic analyses demonstrated significantly higher levels of glutamate and GABA in lung tumors from tumor-bearing mice relative to sham-treated controls. Clinical measurements indicated that glutamate and GABA were markedly increased in SCLC patient plasma, suggesting a systemic elevation of these neurotransmitters associated with tumor progression. Furthermore, pharmacological inhibition of GABA_A_ receptors with flumazenil significantly suppressed tumor growth *in vivo*.

**Conclusions:**

Primary SCLC is associated with cell-type-specific brain transcriptional remodeling and elevated circulating glutamate, providing descriptive evidence for a lung–brain metabolic axis. However, direct causal links within this axis remain to be established through future interventional studies.

## Introduction

Small-cell lung cancer (SCLC) is a highly aggressive neuroendocrine malignancy characterized by rapid progression, early systemic spread, and poor overall survival (1–3).

Historically, SCLC has been classified based on lineage-defining transcription factors, including *ASCL1*, *NEUROD1*, *POU2F3*, and *YAP1 (*4, 5). Emerging evidence indicates that these subtypes correspond to dynamic cell states rather than stable identities, consistent with the pronounced lineage plasticity of SCLC across a continuum of differentiation. For example, *MYC* activation drives a stepwise transition from *ASCL1^+^* neuroendocrine cells through *NEUROD1^+^* neuron-like states toward *YAP1*^+^ mesenchymal-like programs (6). This lineage fluidity reflects a close molecular and functional coupling between SCLC and the nervous system. Neural activity has been shown to modulate glioma progression through both paracrine signaling and neuron–glioma synaptic communication (7), and has also been found in extracranial tumors, including cancers of the prostate, stomach, colorectal region, head and neck, pancreas, breast, and skin (8–19).

Emerging studies indicate that the nervous system actively regulates SCLC progression rather than acting as a passive bystander. Locally, neural innervation serves as a critical determinant of tumor growth and metastatic dissemination; vagotomy effectively inhibits primary tumor formation and restricts distant metastasis (20). In addition, SCLC-N-type and SCLC-A-typetumor cells acquire intrinsic electrical excitability, enabling the long-range propagation of cholinergic *Ca²^+^*waves and the formation of a self-sustaining, autoregulatory signaling network. This elevated electrical activity raises the cellular energy requirements, shifts metabolic reliance toward mitochondrial oxidative phosphorylation, and reveals specific metabolic vulnerabilities (21). Notably, similar to gliomas, SCLC cells can form functional synapses with neurons and receive glutamatergic and GABAergic inputs, which paradoxically promote tumor growth. Activated tumor cells, in turn, enhance neuronal excitability, forming a positive feedback loop—an interaction paradigm first observed in peripheral solid tumors (22). By secreting factors such as reelin to recruit astrocytes and establish a supportive, developmental-like niche, SCLC remodels the neural microenvironment during brain metastasis to facilitate colonization (23). Importantly, therapeutic targeting of these neuro-integrative programs has shown promising preclinical potential (24–26).

Despite these advances, existing studies have primarily focused on local neuro-tumor interactions or brain metastasis. A fundamental and unresolved question remains: can primary SCLC remotely reconfigure the brain? Recent pan-cancer analyses have demonstrated that peripheral tumors can reprogram brain function through neuroendocrine-immune axes (27). For example, pancreatic cancer induces inflammatory remodeling of the hypothalamic microenvironment, driving cachexia-associated metabolic dysregulation (28); peripheral inflammatory cytokines such as IL-6 can access the brain and drive the hyperactivation of neural circuits, including area postrema (AP) neurons, thereby promoting tumor proliferation (29); conversely, central emotional circuits can directly modulate peripheral tumor growth via sympathetic signaling (30). Furthermore, studies have also revealed that during infections such as pneumonia, inflammatory signals from the lungs can be transmitted to specific brain regions, including the hypothalamus, via ascending sensory neural pathways, triggering a series of sickness-related symptoms (31). Collectively, these findings support a new conceptual framework in which tumors hijack and rewire brain–body networks to disrupt systemic homeostasis. Yet, it remains unknown whether a similar lung–brain axis exists in SCLC.

In this study, we employed an *Rb1/Trp53/Myc*–driven SCLC mouse model and conducted whole-brain single-nucleus RNA sequencing to comprehensively and unbiasedly characterize transcriptional reprogramming across all major brain cell types. We found that, even in the absence of direct tumor infiltration, SCLC induces broadly increased *Gad2* expression in basal ganglia cells and GABAergic inhibitory neurons, accompanied by cell-type-specific glial remodeling, including marked downregulation of key myelination-associated genes (*Mbp*, *Mobp*, and *Plp1*). These findings suggest that primary lung tumors may be associated with remote alterations in the brain microenvironment. Further correlative analyses suggest that lung–brain communication may be associated with a glutamate–GABA metabolic axis, though causal evidence remains to be established. In conclusion, our study establishes the first whole-brain single-cell transcriptomic atlas associated with primary small-cell lung cancer (SCLC). The data demonstrate that SCLC is associated with systemic alterations in brain biology, implicating long-range lung–brain signaling and broadening the scope of the lung–brain axis. These results offer a comprehensive biological framework for future therapeutic interventions centered on the systemic impact of the disease.

## Methods

### Animals

We used *Rb1*^fl/fl^*Trp53*^fl/fl^*Myc*^LSL/LSL^ (RPM) female mice, which are available from The Jackson Laboratory (Stock No. 029971). All female mice were maintained and handled in accordance with institutional guidelines approved by the Institutional Animal Care and Use Committee (IACUC).Viral infection experiments were conducted in a biosafety level 2+ (BSL-2+), following the guidelines of the Institutional Biosafety Committee. Aged 6-8-week-old mice were intratracheally instilled with Ad5-Cgrp-Cre adenovirus (GeneChem, China) at a concentration of 1×10 (8) PFU/ml in a total volume of 100 µl per mouse.

### *In vivo* GABA_A_ receptor antagonist experiment

Flumazenil was purchased from MedChemExpress (MCE; catalog no. HY-B0009). In the RPM mouse model, tumor initiation was induced by intratracheal administration of Ad5-Cgrp-Cre adenovirus (GeneChem, China) at a concentration of 1×10 (8) PFU/ml in a total volume of 100 µl per mouse. One week after tumor induction, mice were treated with flumazenil by intraperitoneal injection at a dose of 50 mg/kg every three days. Tumor growth was monitored by *in vivo* bioluminescence imaging at 7 and 9 weeks after tumor induction.

### Statistical analysis and reproducibility

No statistical methods were used to predetermine sample size; however, the sample sizes used are comparable to those reported in previous studies employing single-nucleus RNA sequencing of brain tissue. No animals were excluded from the analyses. Data collection and analysis were not performed blind to the experimental conditions. Specific quality control metrics and exclusion criteria for individual nuclei are described in the data processing section below.

### Single-nucleus RNA sequencing

To minimize variability associated with differences in estrous cycle status, three biological replicates were collected per group. Nuclei were isolated using the Nuclei PURE Prep Kit (Millipore Sigma) according to the manufacturer’s protocol with minor modifications: brain tissue was dissected, rinsed in ice-cold PBS, and transferred into a chilled Dounce homogenizer containing 5 ml of lysis buffer. Nuclei from frozen tissues were isolated using 10K Genomics nuclei isolation buffer with dounce homogenizer. The single nuclei suspensions were counted using luna™ cell counter(Logos Biosystems), appropriate amount of nuclei were loaded onto a microfluidic chip of 10K Genomics 3’ single nuclei RNA kit v1.1, along with reverse transcription reagents, barcoding gel beads and droplet generation oil. The microfluidic chip was run on the 10K Genomics-Perseus™ single cell processing system to generate droplet emulsion, which is transferred to a thermal cycler to perform in drop reverse transcription. Barcoded cDNA was then purified from the emulsion using SPRI beads and PCR amplified. Sequencing libraries were prepared according to the manufacturer’s instructions of 10K Genomics Library Amp Kit and sequenced on an MGI-T7 system in paired-end mode.

### Single nucleus RNA-seq data processing

Raw sequencing reads were processed using the CellCosmo pipeline with default parameters. FASTQ files generated from the MGI T7 platform were aligned to the mouse reference genome (GRCm38) using the STAR algorithm. Gene–barcode matrices were generated for each sample by counting unique molecular identifiers (UMIs) and filtering non-nucleus–associated barcodes. The resulting matrices were imported into the Seurat R toolkit (v4.3.0) for quality control and downstream analysis. All Seurat functions were run with default parameters unless otherwise specified. Low-quality nuclei were filtered based on three quality control metrics: (1) number of detected transcripts (UMIs); (2) number of detected genes (features); and (3) the percentage of reads mapping to mitochondrial genes. The percentage of mitochondrial gene expression was calculated using the PercentageFeatureSet function. Nuclei with fewer than 200 detected features were excluded. For samples with standard sequencing depth, nuclei with more than 3,000 detected features were removed, whereas for samples with higher sequencing depth, the upper threshold was increased to 7,500 features. Nuclei with more than 10% mitochondrial reads were excluded. Data were normalized using the NormalizeData function, and highly variable genes were identified while accounting for the relationship between mean expression and variability.

To correct for batch effects across samples, we applied the Harmony algorithm (v1.0) to the top 30 principal components after scaling and PCA. Harmony was run with group.by.vars = “orig.ident” and default parameters. The Harmony-corrected embeddings were then used for graph-based clustering with FindNeighbors and FindClusters (resolution = 0.5). For two-dimensional visualization, both t-distributed stochastic neighbor embedding (t-SNE) and Uniform Manifold Approximation and Projection (UMAP) were generated using the Harmony-corrected principal components. Potential doublets were identified and removed using DoubletFinder (v2.0.3) with a doublet formation rate of 7.5% and an optimal pK value selected via parameter sweep; nuclei classified as doublets were excluded from downstream analyses.

**Figure 1:**
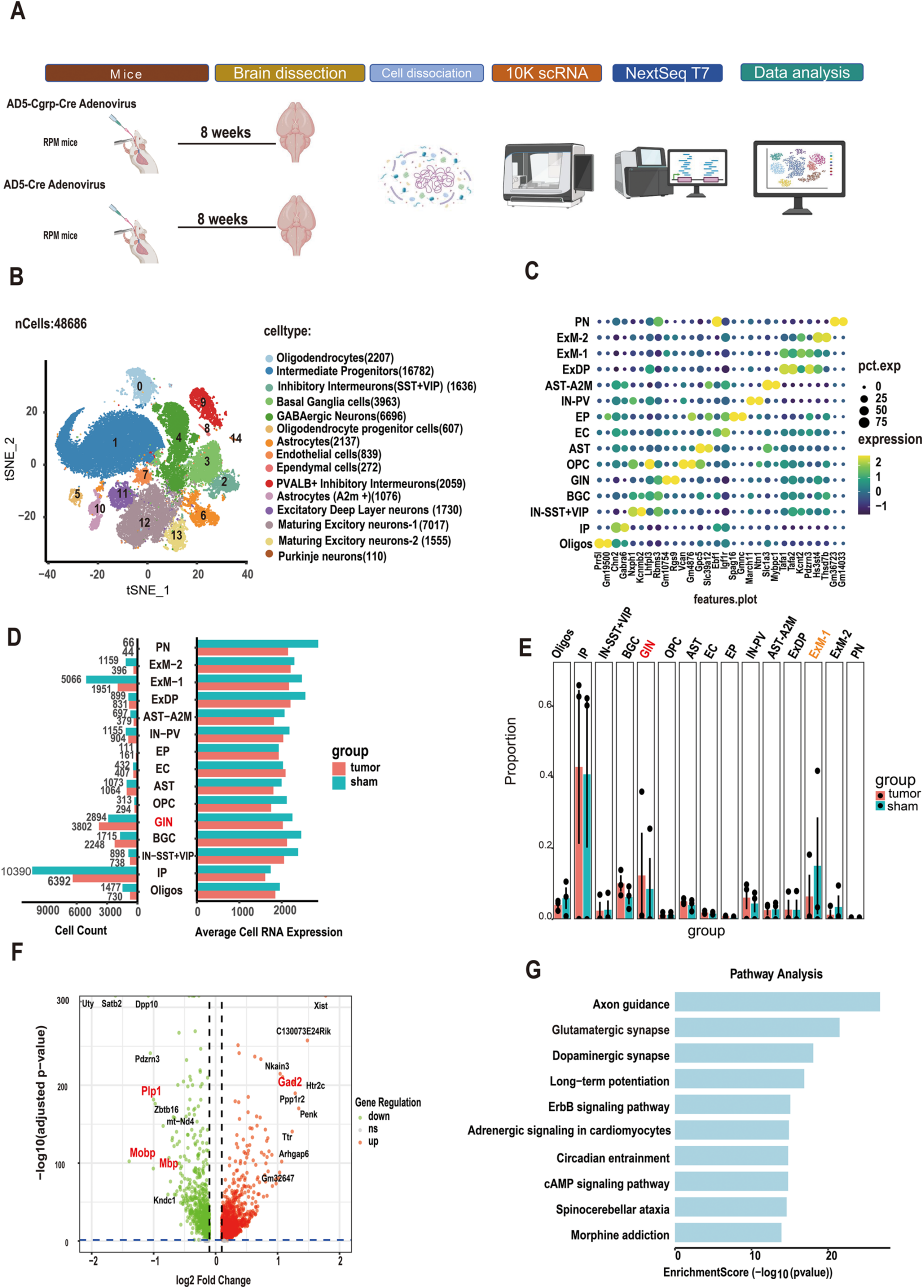
Cell type identification in whole-brain Single-nucleus RNA sequencing (snRNA-seq). Schematic illustrating the experimental workflow for single-nucleus isolation and sequencing from the whole-brain tissues of tumor-bearing mice(tumor) and sham-treated controls(sham) **(A)**. T-Distributed stochastic neighbor embedding (t-SNE) projection of 48,686 single-nucleus transcriptomes derived from the whole brains of three tumor-bearing mice(tumor) and sham-treated controls(sham), with clusters color-coded and annotated *post hoc* based on transcriptional signatures **(B)**. Dot plot highlighting the expression patterns of cell-type-specific markers across clusters; dot size represents the percentage of cells expressing the gene, while color intensity indicates the average expression level **(C)**. Bar plots showing the total cell counts and average RNA counts per cell for each subpopulation in tumor and sham groups **(D)**. Relative proportions of the major brain cell subpopulations in tumor versus sham groups **(E)**. Volcano plot displaying differentially expressed genes across all nuclei from tumor and sham whole-brain samples **(F)**. Genes with significant differential expression (adjusted *P* < 0.05, |log_2_FC| > 0.1) are highlighted in purple; for visualization, *P* values reaching the computational limit of zero were assigned a minimal non-zero value. Differential expression analysis was performed using MAST, incorporating random effects for sample origin and sequencing depth, with *P* values adjusted via Bonferroni correction. GSEA–KEGG enrichment plot illustrating pathway-level alterations between SCLC and RPM whole-brain transcriptomes **(G)**.

In total, 48,686 nuclei were retained and clustered, resulting in the identification of 15 distinct clusters (**Figure 1B**). Among these, 23,808 nuclei under SCLC conditions were included in downstream analyses. Metadata related to nuclei counts, UMI counts, and gene numbers per sample are provided in **Supplementary Figure 1**. Cluster identities were assigned using the FindAllMarkers function with default parameters to identify marker genes for each cluster. Clusters were annotated based on canonical cell-type markers. Differential gene expression analysis was performed using MAST (v1.18.0), accounting for sequencing depth and sample origin. Genes with an adjusted *P* value < 0.05 and an absolute log_2_ fold change > 0.1 were considered significantly differentially expressed.

### Transcriptome sequencing and analysis

Lung tissues were flash-frozen in liquid nitrogen and ground into powder. Total RNA was extracted with TRIzol Reagent (Invitrogen, USA) according to the manufacturer’s instructions. RNA purity and concentration were measured with a NanoDrop 2000 spectrophotometer (Thermo Fisher Scientific), and integrity was assessed using an Agilent 2100 Bioanalyzer; only samples with RIN ≥ 7 were used for library preparation.

RNA sequencing libraries were constructed using the NEBNext Ultra II RNA Library Prep Kit (NEB #E7770), with poly(A) selection for mRNA enrichment. Fragment size distribution was assessed using the Agilent 2100 Bioanalyzer, and paired-end sequencing (2 × 150 bp) was performed on the Illumina NovaSeq 6000 platform.

Raw reads were quality-controlled using fastp to remove adaptors and low-quality sequences. Clean reads were aligned to the mouse reference genome (GRCm38/mm10) using STAR aligner (v2.7.3a). Gene-level counts were calculated with featureCounts. Differential expression analysis was performed using DESeq2 (v1.30.1) in R, with thresholds set at |log_2_FC| > 1 and FDR < 0.05. GO and KEGG pathway enrichment analyses were conducted on differentially expressed genes using the clusterProfiler R package. Data visualizations included heatmaps (pheatmap), volcano plots (EnhancedVolcano), and PCA plots.

### LC-MS/MS-based quantification of neurotransmitters

LC-MS/MS-based quantification of neurotransmitters was performed using the following procedures. Neurotransmitter standards, including L-histidine, picolinic acid, tyramine, acetylcholine chloride, hydroxytyramine hydrochloride, serotonin hydrochloride, adrenaline hydrochloride, kynurenic acid, levodopa, xanthurenic acid, DL-kynurenine, and 5-hydroxytryptophan, were purchased from Sigma-Aldrich (Shanghai, China). L-glutamine, histamine, tryptamine, 5-hydroxyindole-3-acetic acid, vanillylmandelic acid, and melatonin were obtained from Aladdin (Shanghai, China). Noradrenaline hydrochloride was purchased from TRC (Toronto, Canada), and L-asparagine was obtained from OKA (Beijing, China). γ-Aminobutyric acid (GABA), L-glutamic acid, L-tyrosine, and L-tryptophan were acquired from Sinopharm Chemical Reagent Co., Ltd. (Shanghai, China). The isotope−labeled internal standard DL-tryptophan-2,3,3-d3 (Trp-d3) was purchased from C/D/N Isotopes Inc. (Pointe-Claire, Canada).

Lung tissues were extracted with precooled 80% methanol (Meiji Bio, China), followed by vortex mixing and ultrasonic disruption. Samples were centrifuged at 4 °C, and the supernatants were collected, evaporated to dryness under nitrogen, and reconstituted in the mobile phase for LC-MS/MS analysis. Primary stock solutions of individual neurotransmitter standards were prepared in methanol or water. A mixed working standard solution was prepared by combining appropriate volumes of each stock solution and diluting with 10% formic acid in methanol/water (1:1, v/v) to generate a series of concentration gradients. The internal standard stock solution of Trp-d3 was prepared at a concentration of 1,000 ng/mL using the same solvent. All stock and working solutions were stored at 4 °C prior to use.

Samples were extracted in 600 μL of 10% formic acid in methanol/water (1:1, v/v). Two steel beads were added, and the samples were vortexed for 30 s, followed by homogenization in a tissue grinder at 55 Hz for 90 s. The homogenates were centrifuged at 12,000 rpm for 5 min at 4 °C. For low-concentration analyte detection, 100 µL of the supernatant was mixed with 100 µL of Trp-d3 internal standard solution (10 ng/mL), vortexed for 30 s, filtered through a 0.22 µm membrane, and transferred to LC−MS vials. For high-concentration analyte detection, an aliquot of the supernatant was diluted 20−fold with 10% formic acid in methanol/water (1:1, v/v) and processed using the same procedure.

The concentrations of analytes in tissue samples were calculated according to the following equations: content = C × 0.6 × 2/Amount; content (20-fold diluted samples) = C ×0.6×2×20/Amount, where content is expressed in μg/g, C represents the measured concentration (ng/mL), and Amount denotes the tissue weight (mg). Data acquisition and processing were performed using LabSolutions or MassHunter software. Statistical differences between groups were assessed using Student’s t-test or the Mann–Whitney U-test, with *P* < 0.05 considered statistically significant.

### Enzyme-linked immunosorbent assay

#### Sample collection

Plasma samples were collected from 50 patients with small cell lung cancer (SCLC) and 10 healthy individuals at the National Center for Respiratory Medicine, National Clinical Research Center for Respiratory Disease, State Key Laboratory of Respiratory Disease, Guangzhou Institute of Respiratory Health, The First Affiliated Hospital of Guangzhou Medical University, Guangzhou. This study was conducted in accordance with the principles of the Declaration of Helsinki and was approved by the Institutional Review Board of The First Affiliated Hospital of Guangzhou Medical University. Written informed consent was obtained from all participants prior to sample collection.

The concentrations of GABA and glutamate in plasma were quantified using commercial Human GABA and Glutamate ELISA Kits (Jiangsu Enzyme Science Biotechnology Co., China), respectively, based on the double-antibody sandwich method according to the manufacturer’s instructions. Briefly, 50 µL of standards were added to the standard wells. For sample testing, 10 µL of plasma and 40 µL of sample diluent were added to the respective wells, resulting in a 5-fold dilution. Subsequently, 100 µL of HRP-conjugate reagent was added to each well, and the microplates were covered and incubated for 60 minutes at 37 °C. Following five rigorous washes with the provided wash buffer, 50 µL of Chromogen Solution A and 50 µL of Chromogen Solution B were added to each well and incubated at 37 °C for 15 minutes in the dark. The reactions were terminated by adding 50 µL of Stop Solution per well. The optical density (OD) was measured at 450 nm using a microplate reader within 15 minutes. All samples were measured in duplicate. Standard curves were generated using a four-parameter logistic regression, and the actual concentrations of GABA and glutamate were calculated by multiplying the curve-derived values by the dilution factor of 5.

#### Statistical analysis

Data analysis was performed using GraphPad Prism5 software. Statistical significance between groups was assessed using unpaired Student’s t-tests or non-parametric tests (Mann-Whitney U test) as appropriate. A p-value of less than 0.05 was considered statistically significant.

### Gene set enrichment analysis

Gene set enrichment analysis was performed using the fgsea package (v1.18.0) with Hallmark and KEGG gene sets from MSigDB v7.2. For each cluster, genes were ranked by log2 fold change from MAST analysis and analyzed with the fgseaMultilevel function using default parameters and a random seed of 1000. Enriched pathways were defined as those with adjusted *P*-values < 0.1. Conversion between mouse and human annotations was performed using biomaRt (v2.48.2).

### Trajectory inference and analysis with Monocle 3/Monocle 2

Trajectory analysis of OPC/Oligos clusters was conducted using Monocle 2 and Monocle 3. Single nuclei were embedded into a two-dimensional space via dimensional reduction, and mutual nearest neighbor alignment was used to remove batch effects. Cells were connected in a semi-supervised manner to build trajectories. For these clusters, Seurat-integrated objects were used directly without additional batch correction or reduction. Trajectory roots were defined programmatically based on nodes enriched for “younger” cells. Trajectories and pseudotime were consistent with the distribution of tumor and sham cells. Differential gene testing along trajectories was performed using Moran’s I test in Monocle 2/Monocle 3, selecting genes with q-values < 0.05 as trajectory-dependent genes.

Functional enrichment of these genes was performed with enrichR (v3.0) across GO Biological Process, Cellular Component, and Molecular Function databases (2018). Only terms with adjusted *P*-values < 0.05 were retained. The top 10 significant GO terms for each module were visualized using dot plots.

### Cellchat analysis

Cell–cell communication was inferred using CellChat (v2.1.7). Single-nucleus transcriptomes from mouse brain tissues were first subset into tumor-bearing (SCLC) and sham-treated control groups. As the mouse ligand–receptor database (CellChatDB.mouse) contains fewer curated interactions than its human counterpart, mouse gene symbols were converted to human orthologs using biomaRt (v2.48.2), and the human database (CellChatDB.human) was applied to maximize coverage of signaling pathways. Overexpressed genes and ligand–receptor interactions were identified, and expression data were projected onto a protein–protein interaction network. Communication probabilities were computed with 1,000 permutations, and significant interactions were filtered at P < 0.1. Only ligand–receptor pairs with inferred directionality were retained for downstream visualization.

## Results

### Single-nucleus profiling reveals brain-wide transcriptional alterations in SCLC

To investigate how small-cell lung cancer (SCLC) influences the brain, we used the *Rb1/Trp53/Myc* (*RPM*) mouse model (25), in which tumors were induced via intratracheal delivery of the Ad5-Cgrp-Cre adenovirus (**Figure 1A****;** **Supplementary Figure 1A**). Tumor formation was confirmed by *in vivo* imaging eight weeks after infection. We subsequently performed single-nucleus RNA sequencing (snRNA-seq) on whole-brain tissues from three tumor-bearing mice and three sham-treated controls.Unsupervised clustering of 48,686 high-quality nuclei identified 15 transcriptionally distinct clusters (**Figure 1B**). Cell types were annotated using canonical marker genes and visualized in a dot plot (**Figure 1C**), revealing a diverse repertoire of neural and non-neural populations, including intermediate progenitors (IPs), oligodendrocyte precursor cells (OPCs), oligodendrocytes (Oligos), astrocytes (AST), *A2m*-high astrocytes (AST-A2M) (26), basal ganglia cells (BGC), endothelial cells (EC), and eight distinct neuronal subtypes.

Further characterization of neuronal subtypes identified clusters 2, 3, 4, and 9 as inhibitory neurons, and clusters 11, 12, and 13 as excitatory neurons. Intriguingly, cluster 3 exhibited a mixed neurotransmitter signature, co-expressing *Slc17a6* (*Vglut2*), *Gad1*, and *Gad2*, consistent with a basal ganglia identity (32). Subtype analysis showed enrichment of *Pvalb* in cluster 9 (PVALB^+^ interneurons), while Sst and Vip were co-enriched in cluster 2. Excitatory neurons were further subdivided into ExDP (Excitatory Deep Layer neurons), ExM-1 (Maturing Excitatory neurons-1), and ExM-2(Maturing Excitatory neurons-2) subtypes based on *Satb2* and *Lmo3* expression patterns.

**Figure 2:**
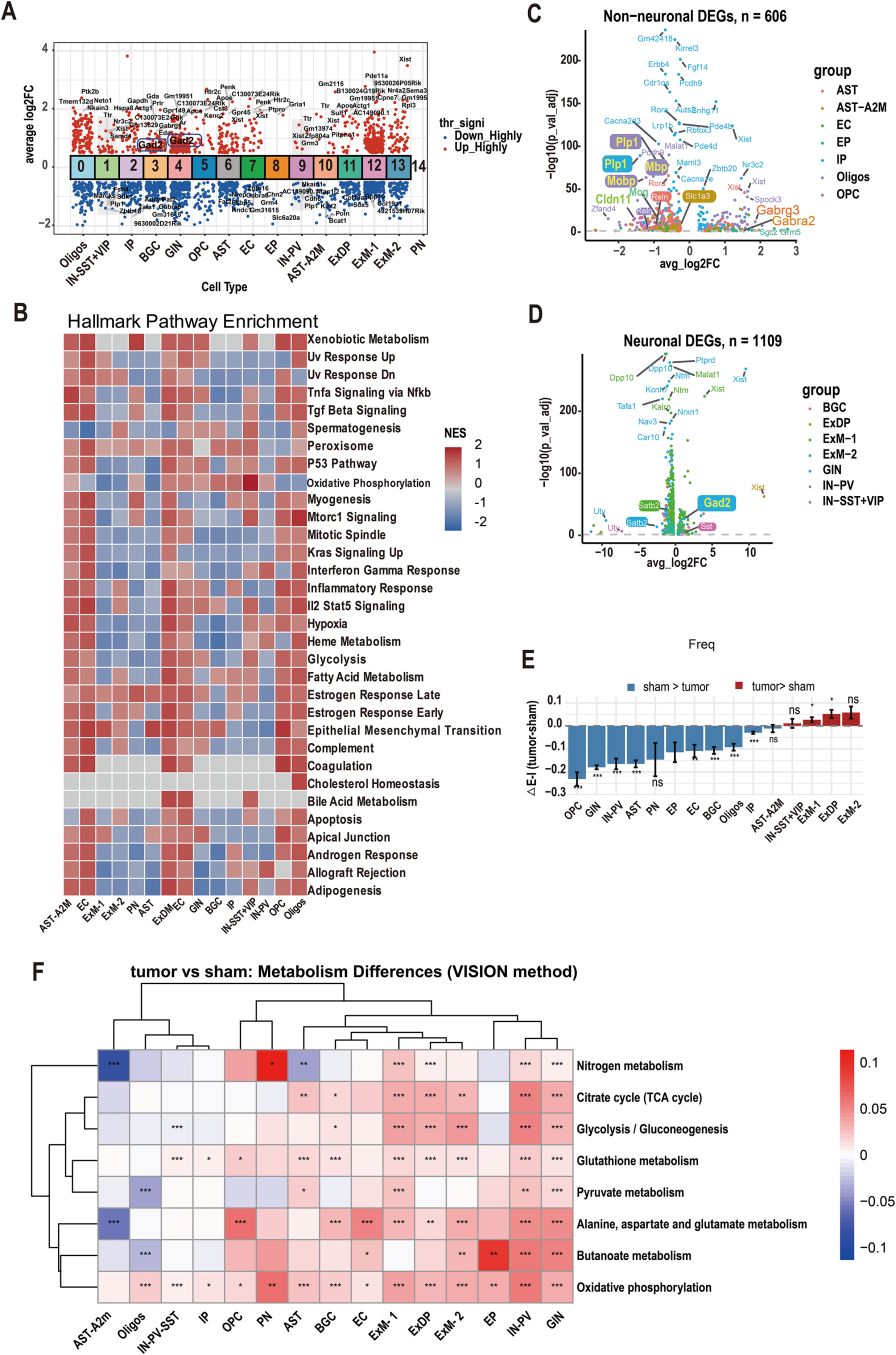
SCLC induces global and cell-type-specific transcriptional and metabolic reprogramming in the brain. Strip plot illustrating differentially expressed (DE) genes across individual cell types. Significant genes (adjusted *P* < 0.05, |log_2_FC| > 0.1, identified via FindMarkers analysis with covariate adjustment are highlighted in color. The top five upregulated and downregulated genes per cluster are labeled; notably, *Gad2* is specifically highlighted across multiple clusters due to its biological significance in this study, regardless of its fold-change ranking **(A)**. Heatmap displaying Hallmark GSEA scores across major brain cell types, color-coded by normalized enrichment score (NES). Pathways with adjusted P < 0.1 were considered significant. Neuronal clusters are enriched for oxidative phosphorylation, while non-neuronal clusters show enrichment for mesenchymal-like transition (MLT, denoted as “EMT” in the Hallmark database) and inflammation-related pathways **(B)**. Volcano plot showing differentially expressed genes within non-neuronal cell subtypes between tumor and sham conditions, highlighting the downregulation of key myelin-related genes, including *Plp1*, *Mbp*, *Mobp*, *Mog*, and *Cldn11* **(C)**. Volcano plot displaying differentially expressed genes in neuronal subtypes, highlighting the notable upregulation of *Gad2* in SCLC **(D)**. Alterations in the excitatory–inhibitory **(E–I)** balance across brain cell populations. Excitatory and inhibitory module scores were calculated using the Seurat AddModuleScore function based on excitatory markers (*Slc17a7, Slc17a6, Slc17a8*) and inhibitory markers (*Gad1, Gad2, Vgat*). The difference in E–I scores (ΔE–I) between tumor and sham conditions is shown for each major cell type, illustrating the shifts in signaling induced by SCLC (Data are presented as mean ± SEM) **(E)**. Single-cell pathway activity inference using Vision, revealing the activation of oxidative phosphorylation, the TCA cycle, and glutamate metabolism-related pathways in diverse neuronal populations, indicating metabolic reprogramming **(F)**.

Analysis of brain cellular composition revealed a reciprocal shift in neuronal populations. GABAergic inhibitory neurons increased in both absolute number and relative proportion (**Figures 1D, E**), whereas the ExM-1 population declined in relative proportion (**Figures 1D, E**). This opposing pattern indicates a shift in the overall excitatory-inhibitory (E/I) balance toward enhanced inhibition in the brains of tumor-bearing mice. We also present the cell subtype counts and proportions for each sample. Notably, two samples across groups Tumor3 and Sham3, exhibited inconsistent relative cell proportions. This discrepancy may be attributed to the high myelin content of adult mouse brains, which renders them exceedingly difficult to digest. During enzymatic dissociation or nuclear extraction across different samples, certain fragile neuronal subpopulations are prone to cell death, resulting in distorted proportions of cells ultimately captured for sequencing (**Supplementary Figure 1B****;** **Figure 2**).

To systematically characterize transcriptional reprogramming across the brain, we performed differential expression analysis using the Model-based Analysis of Single-cell Transcriptomics (MAST) with sample origin modeled as a random effect. Compared with sham-treated controls, tumor-bearing mice exhibited widespread transcriptional alterations, including 434 upregulated and 249 downregulated genes (padj < 0.05, |log_2_FC| > 0.1) (**Figure 1F**). Among the most prominent changes was increased expression of *Gad2* (*Glutamate decarboxylase 2*), which encodes the rate-limiting enzyme responsible for GABA synthesis. In contrast, a suite of genes critical for myelin formation, including *Mbp*, *Plp1*, and *Mobp*, were reduced (**Figure 1F**). This reflects a multi-faceted neurological impact of SCLC, encompassing both functional neurotransmission and structural myelin integrity. Subsequently, gene set enrichment analysis further highlighted significant enrichment of axon guidance, GABAergic synapses, and glutamatergic synapses pathways, suggesting that SCLC induces remodeling of the neuronal signaling networks and active restructuring of neural circuit architecture (**Figure 1G**).

### SCLC induces global and cell-type-specific transcriptional and metabolic reprogramming in the brain

To systematically delineate the brain-wide transcriptional impact of SCLC, we performed differential expression analysis across all major cell populations, revealing widespread gene expression changes spanning both neuronal and non-neuronal compartments (**Figure 2A**). *Gad2* expression broadly increased in basal ganglia cells (BGC) and GABAergic inhibitory neurons (**Figure 2A**). Previous studies have reported that GAD2 is most frequently observed in several neuroendocrine tumors, particularly neuroendocrine carcinoma (58.3%), pancreatic neuroendocrine tumors (63.2%), granular cell tumors (37.0%) and pulmonary neuroendocrine tumors (11.1%), with lower prevalence across other tumor types, suggesting GAD2 expression may be associated with neuroendocrine features (33). Collectively, these changes reflect a broad molecular response of the brain’s neurochemical landscape to peripheral tumor burden, involving multiple cell populations.

Gene Set Enrichment Analysis using Hallmark pathways revealed distinct, cell-type-specific responses (**Figure 2B**). Neuronal populations showed enrichment of oxidative phosphorylation pathways, whereas non-neuronal populations exhibited increased signatures associated with mesenchymal-like transition (MLT; corresponding to the Hallmark EMT gene set but interpreted here as a mesenchymal-activation program given the non-epithelial origin of these cells) and inflammatory signaling. Astrocytes and A2M^+^ astrocytes showed significant enrichment in inflammatory response, IL2-STAT5 signaling, MLT, and TNFA signaling via NF-κB, consistent with inflammatory activation and migratory remodeling. Endothelial cells showed enrichment of MLT, coagulation, and IL6-JAK-STAT3 signaling, indicating vascular remodeling and altered barrier function. OPCs and oligodendrocytes were enriched for hypoxia, MLT, and TNFA signaling, suggesting activation of white matter injury responses. Notably, MLT-related signatures were detected across the majority of brain cell populations, implying broad phenotypic adaptation to tumor-derived signals (34)(**Figure 2B**).

Consistent with these pathway changes, analysis of non-neuronal populations revealed significant downregulation of key myelin-associated genes, including *Plp1, Mbp, Mobp, Mog, and Cldn11*(**Figure 2C**). In neuronal populations, *Gad2* remained one of the most consistently upregulated genes (**Figure 2D**). To further assess functional changes in neural network balance, we calculating excitatory and inhibitory module scores using the AddModuleScore function, based on canonical excitatory markers (*Slc17a6/7/8*) and inhibitory markers (*Gad1, Gad2, Vgat*). Cells were categorized into excitatory, inhibitory, mixed, and non-excitatory/non-inhibitory neutral groups based on module enrichment scores, which closely aligned with the established E–I identities revealed in our dataset (**Supplementary Figure 1C**). Across multiple neuronal and non-neuronal populations, SCLC induced a marked increase in inhibitory signatures accompanied by reduced excitatory markers (**Figure 2E****;** **Supplementary Figure 1D**), indicating a global shift in excitatory–inhibitory balance. Indicating a brain-wide shift toward increased GABAergic transcriptional potential.

Specifically, we found that oligodendrocyte progenitor cells (OPCs), GABAergic inhibitory neurons (GINs), *PVALB+* Inhibitory interneurons (IN-PV), astrocytes (AST), endothelial cells (EC), oligodendrocytes (Oligos), ependymal cells (EP), basal ganglia cells (BGC), and intermediate progenitors (IP) all displayed a shift toward inhibitory marker enrichment following tumor growth, supporting the notion of a brain-wide bias toward inhibition. (**Figure 2E****;** **Supplementary Figure 1D**). Such dysregulation of brain excitatory–inhibitory (E/I) balance is commonly associated with neural circuit dysfunction, suggesting that peripheral tumors may be associated with remote alterations in brain network stability. Complementary single-cell pathway inference approaches further supported these findings. Single-cell pathway activity inference using Vision further revealed a pronounced neuronal metabolic reprogramming, characterized by the activation of oxidative phosphorylation, the TCA cycle, and glutamate metabolism–related pathways (**Figure 2F**). Concurrently, CytoTRACE analysis identified ExM-1, GINs, and ExM-2 as relatively less differentiated populations, suggesting that these populations retain higher developmental potential (**Supplementary Figures 2A, B**). In parallel, metabolic flux inference using METAFlux demonstrated increased glucose uptake and glycolytic activity across multiple populations, particularly in BGC, ExM-1, and GINs, consistent with enhanced metabolic demand in the tumor-associated brain environment (**Supplementary Figure 2G**).

### SCLC disrupts oligodendroglial development and myelination-associated networks

**Figure 3:**
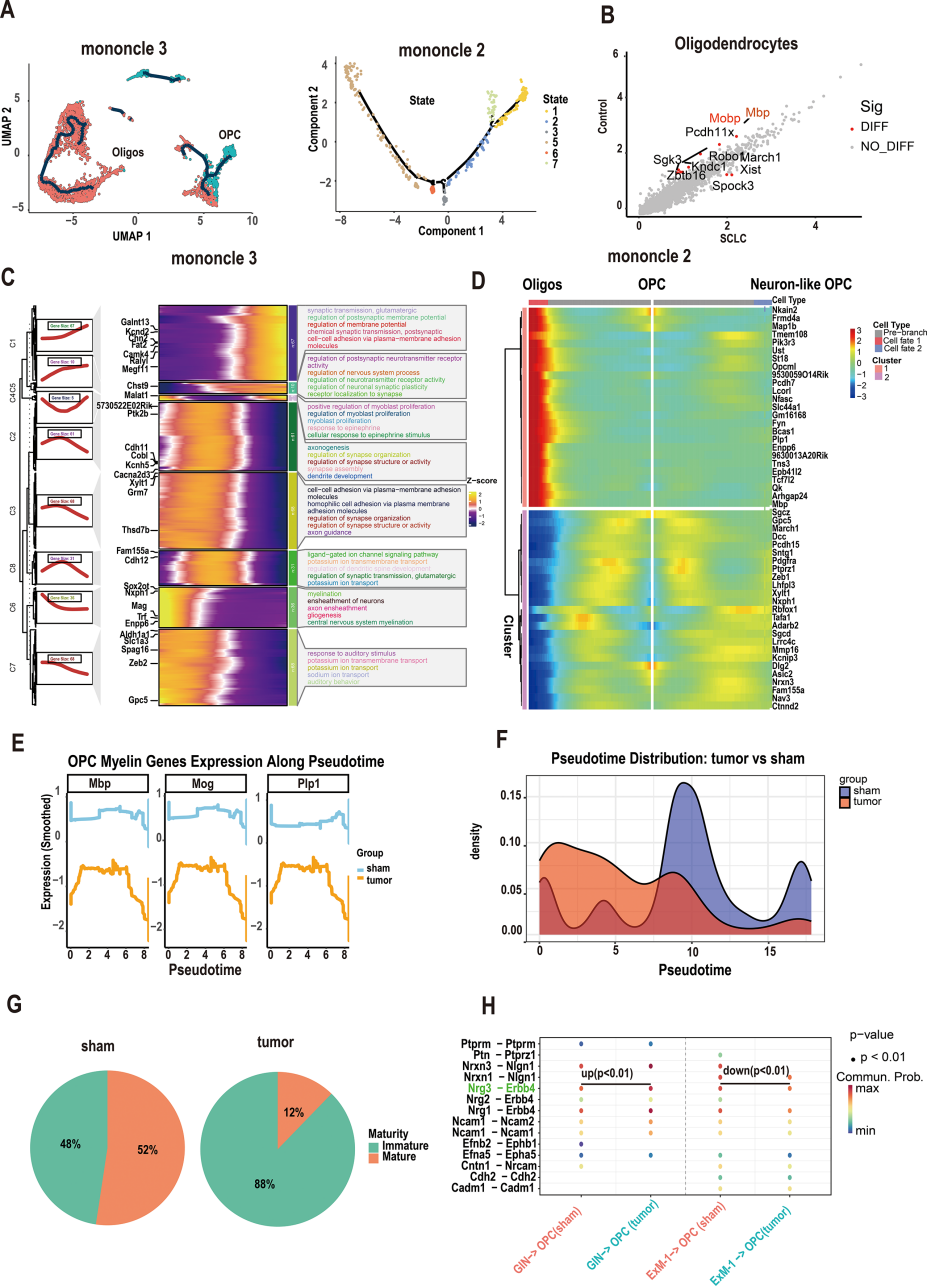
SCLC interrupts the oligodendrocyte lineage trajectory of OPCs. Pseudotemporal ordering using Monocle 2 and Monocle 3 characterizing the lineage transition of oligodendrocyte progenitor cells (OPCs) toward mature oligodendrocytes **(A)**. Scatter plot displaying differentially expressed (DE) genes in oligodendrocytes between sham and tumor mouse brains **(B)**. Heatmaps showing modules of trajectory differentially expressed genes (t-DEGs) along the oligodendrocyte developmental lineage, as identified via Monocle 3 **(C)** and Monocle 2 **(D)** analysis. Kinetics plots illustrating the relative expression of key markers (*Mbp*, *Plp1*, and *Mog*) along the pseudotime trajectory **(E)**. Proportional analysis of OPCs transitioning into mature oligodendrocytes versus “neuron-like” OPCs, highlighting a significant reduction in mature differentiation under SCLC conditions **(F)**. Distribution of cells along the pseudotime timeline, comparing the density and proportions of developmental stages between sham and tumor conditions **(G)**. Differential cell–cell interactions between ExM-1, GINs, and OPCs in SCLC versus sham groups inferred by CellChat; red indicates upregulated and blue indicates downregulated interaction strengths **(H)**.

Microglia, the resident macrophage-like cells of the brain, serve as key mediators of immune surveillance, performing functions such as cytokine secretion, antigen presentation, and phagocytic clearance. In the context of SCLC, we observed a marked downregulation of *Mbp* within the oligodendrocyte lineage. Given that *Mbp* is essential for maintaining myelin structural integrity, its reduced expression is indicative of impaired myelination or progressive demyelination. Such myelin instability is known to activate microglia and promote pro-inflammatory cytokines (e.g., IL-6 and TNF-α), fostering a chronic neuroinflammatory environment. Neuronal activity has been reported to influence myelination (35, 36). Oligodendrocyte precursor cell (OPC)-to-oligodendrocyte differentiation is characterized as a sequential five-stage process: proliferation, migration, adhesion to axons, maturation with synthesis of myelin structural proteins (PLP1, MBP, MOG), and axonal myelin wrapping (37). Within this developmental framework, we hypothesized that the myelin deficits observed in tumor-bearing mice are not primarily due to a loss of mature oligodendrocytes, but instead arise from impaired OPC-to-oligodendrocyte differentiation. To test this, we reconstructed OPC differentiation trajectories using Monocle 2 and Monocle 3. In sham-treated control brains, OPCs followed a well-defined and continuous trajectory toward mature oligodendrocytes (**Figure 3A**). Several lines of evidence supported disrupted maturation in the SCLC group. First, differential expression analysis revealed widespread downregulation of myelination-related genes, including *Mbp*, *Plp1 and Mog*, in oligodendrocytes (**Figure 3B****;** **Supplementary Figures 3A–F**). Second, pseudotime-based heatmap analyses identified multiple developmental states during OPC maturation, including canonical OPCs and neuron-like OPCs, accompanied by dynamically regulated gene modules across the differentiation process (**Figures 3C, D**). Third, during the transition from OPCs to oligodendrocytes, expression of maturation-associated genes such as *Plp1 and Mog*, were consistently expressed at lower levels in tumor-bearing mice compared with sham-treated controls (**Figure 3E**). Finally, quantitative analysis demonstrated accumulation of immature oligodendrocytes, indicating delayed progression along the differentiation trajectory (**Figures 3F, G**).

Given that early OPC proliferation and differentiation are regulated by NRG-ERBB4 signaling (38, 39), we next examined intercellular communication using CellChat. In brains of tumor-bearing mice, OPCs exhibited increased predicted ligand-receptor-based signaling interactions with GABAergic inhibitory neurons (GINs) and reduced interactions with excitatory neurons (ExM-1) (**Figure 3H**). This bimodal signaling pattern was associated with distinct functional consequences. Specifically, reduced NRG–ERBB4 signaling from excitatory neurons restricts NMDA receptor-dependent myelin expansion, contributing to myelin loss. Conversely, NRG-ERBB4 signaling from inhibitory neurons was increased, consistent with enhanced inhibitory neuronal input to OPCs, which has been reported to be associated with reduced myelination (38, 39). Collectively, these findings demonstrate that OPC-to-oligodendrocyte maturation is impaired in SCLC, driven by an imbalanced integration of excitatory and inhibitory signals, with excessive inhibitory input acting as a primary driver of myelination failure.

To further define the transcriptional architecture underlying myelin deficits, we performed Weighted Gene Co-expression Network Analysis (WGCNA). Five distinct transcriptional modules were identified, with Module 2 showing a remarkably strong positive correlation with SCLC status (**Supplementary Figure 5A**). Cell-type enrichment analysis further revealed that this module was enriched in OPCs and oligodendrocyte (**Supplementary Figure 5B**) and contained core myelination genes such as *Mbp* and *Plp1*, as central network hubs (**Supplementary Figures 5C, D**). Taken together, our data indicate that the myelin loss observed in brain under SCLC results from a global, glia-centered disruption of coordinated gene expression programs rather than isolated transcriptional changes. Given that myelination is fundamentally dependent on intercellular signaling, we next investigated whether these observed deficits were accompanied by alterations in the cellular communication landscape. Signaling pattern clustering revealed distinct input and output profiles specific to each cell type (**Supplementary Figures 5E, F**). Comparative communication analysis between the tumor-bearing mice and sham-treated controls showed a widespread attenuation of pathways important for glial homeostasis, including *FGF, CLDN, EPHA, and MAG* signaling (**Supplementary Figure 5G**). Among these, the *CLDN* (Claudin) pathway, which supports both blood-brain barrier integrity and myelination, was markedly reduced. The corresponding network map further illustrates altered glial-vascular interactions in tumor-bearing brains (**Supplementary Figure 5H****).** Together, these data indicate that SCLC is associated with defective oligodendrocyte lineage progression accompanied by coordinated disruption of myelination gene networks and intercellular signaling pathways required for glial maturation and function.

### Small-cell lung cancer is associated with expansion of a distinct brain endothelial subset and altered BBB-related gene expression

Brain vascular endothelial cells form the structural backbone of the blood-brain barrier (BBB) and represent a critical interface between the peripheral circulation and the central nervous system. Given their role in maintaining neural homeostasis, we examined whether primary SCLC induces vascular remodeling that could contribute to long-range brain alterations.

**Figure 4:**
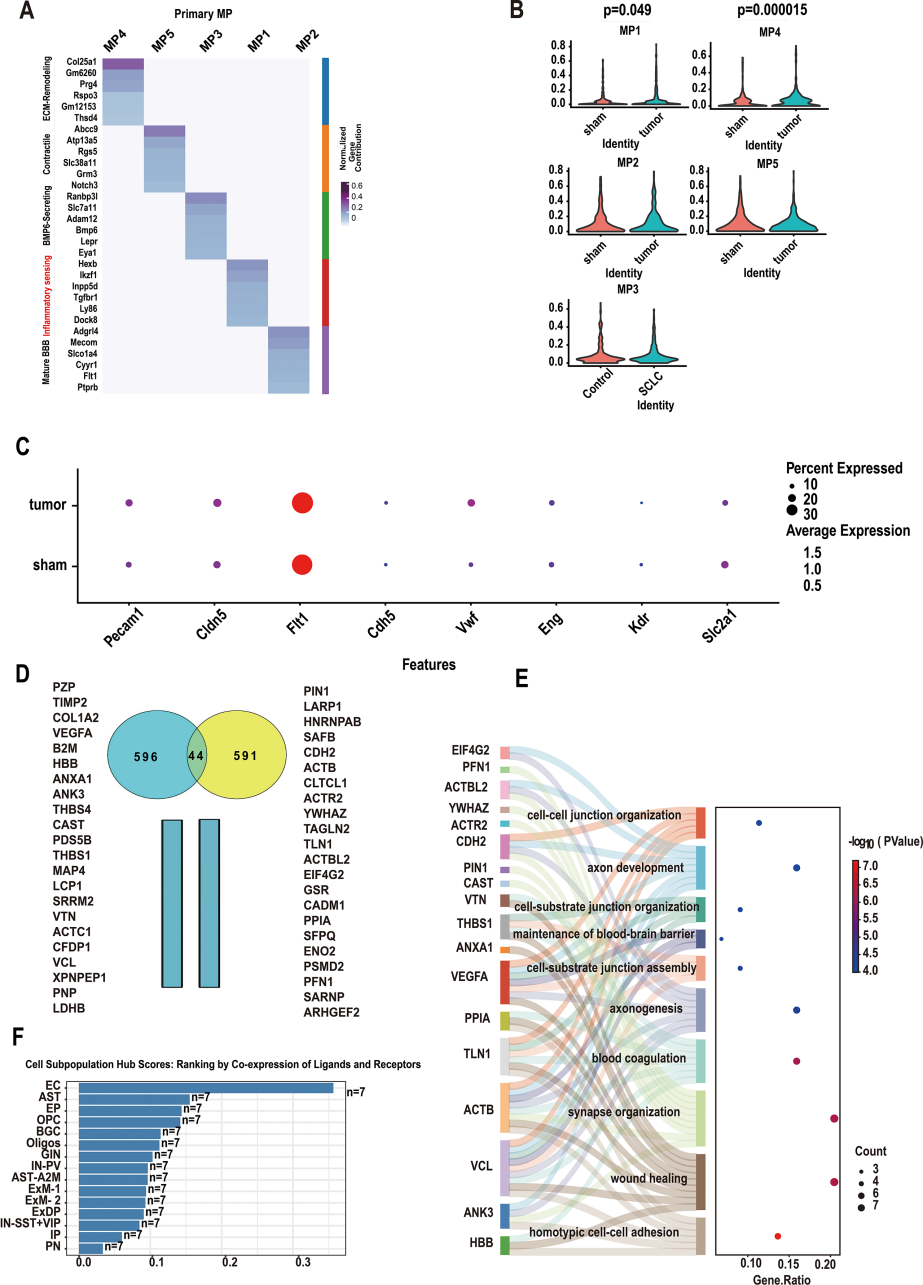
SCLC induces inflammatory reprogramming of brain endothelial cells and enhances predicted endothelial–neuronal signaling interactions. Non-negative matrix factorization (NMF) defines five endothelial metaprograms: MP1, inflammatory sensing-EC; MP2, mature BBB-EC; MP3, BMP6-secreting-EC; MP4, ECM-remodeling-EC; and MP5, contractile-EC **(A)**. Violin plots show differential activity of MP1–MP5 between sham and SCLC conditions, with MP1 and MP4 upregulation consistent with stress-associated endothelial inflammation and ECM remodeling **(B)**. Intersection analysis of secreted proteins from human small-cell lung cancer (SCLC) samples and human cell lines identified 44 SCLC-specific secreted proteins, human serum and cell line secretome data were obtained from Supplementary Datasets S1–S3 of Fahrmann et al. (Cancers 2021, 13, 3972) **(C)**. GO-BP analysis of the 44 SCLC-specific secreted proteins identified in panel**(D)**.Endothelial cells exhibit high expression of Flt1 (VEGFR1), identifying an inflammatory endothelial subtype with significantly higher expression in tumor-bearing mice(tumor) and than in sham-treated controls(sham) **(E)**.

To systematically delineate endothelial functional states, we employed Non-negative Matrix Factorization (NMF) to classify endothelial cells into five distinct metaprograms (MPs): inflammatory sensing-EC (MP1), mature BBB-EC (MP2), BMP6-secreting-EC (MP3), ECM-remodeling-EC (MP4) and contractile-EC (MP5) (**Figure 4A**). In SCLC brains, MP1 and MP4 populations were significantly expanded, indicating vascular stress and structural remodeling (**Figure 4B****;** **Supplementary Figure 6A**). Notably, both MP1 and MP4 displayed attenuated immune responses and diminished ECM-supportive functions. CellChat analysis further predicted ligand-receptor-mediated increased NRG–ERBB4 signaling from inhibitory GABAergic neurons to endothelial cells, alongside reduced input from excitatory neurons (**Supplementary Figures 6B, C**). These findings suggest that endothelial cells may participate in the remodeling of neuronal-vascular signaling interactions rather than acting solely as passive responders to tumor-derived signals.

To identify circulating factors potentially mediating these vascular changes, we intersected proteomic data from sera of SCLC patients with the secretome profiles from human SCLC cell lines(H1607, HCC4002, H209, H211, H2195, H2679, H345, H524,H526, H69P, H69AD, H82, HCC4001, HCC4003, HCC4004, HCC4005, H1048).Both datasets were obtained from the supplementary materials of Fahrmann et al. (2021) (40), specifically Dataset S1 (serum protein abundance in early-stage SCLC cases versus controls), Dataset S3 (pre-diagnostic serum proteomic profiles), and Dataset S2 (secretome profiles of the 17 SCLC cell lines).Intersection of these datasets yielding 44 candidate factors enriched in the tumor secretory profile. Gene ontology analysis linked these factors to BBB regulation, inflammation, and signal transduction (**Figures 4C, D**). Further investigation revealed that brain endothelial cells displayed the highest ligand-receptor co-expression score among brain cell types, supporting their central role in mediating systemic tumor signals (**Supplementary Figure 6D**). Among these candidate factors, VEGFA was notably elevated. Given that increased circulating VEGFA is a well-established biomarker of disrupted blood–brain barrier (BBB) (41, 42), and its receptor Flt1 (Vegfr) is highly expressed in brain endothelial cells, these findings provide additional support for BBB dysfunction in the SCLC setting (**Figure 4E**). Consistent with this interpretation, VEGF-mediated disruption of endothelial tight-junction integrity has been shown to promote blood–brain barrier breakdown, and VEGF-related blood–brain barrier dysfunction has also been implicated in stress-associatedneurovascular alterations (43, 44).

### Multi-omics-based correlative analysis suggests a potential metabolic link in the SCLC lung-brain axis: the role of glutamate/GABA metabolic reprogramming

Collectively, our data indicate extensive remodeling of the brain microenvironment in SCLC, characterized by coordinated transcriptional shifts across multiple cell populations, including altered neuronal excitatory–inhibitory balance and impaired oligodendrocyte maturation. These changes suggest a systemic response to distal tumor burden rather than stochastic local variation. Based on these observations, we hypothesize the existence of a lung–brain metabolic axis in which tumor-associated alterations in neurotransmitter metabolism may contribute to long-range signaling.

**Figure 5:**
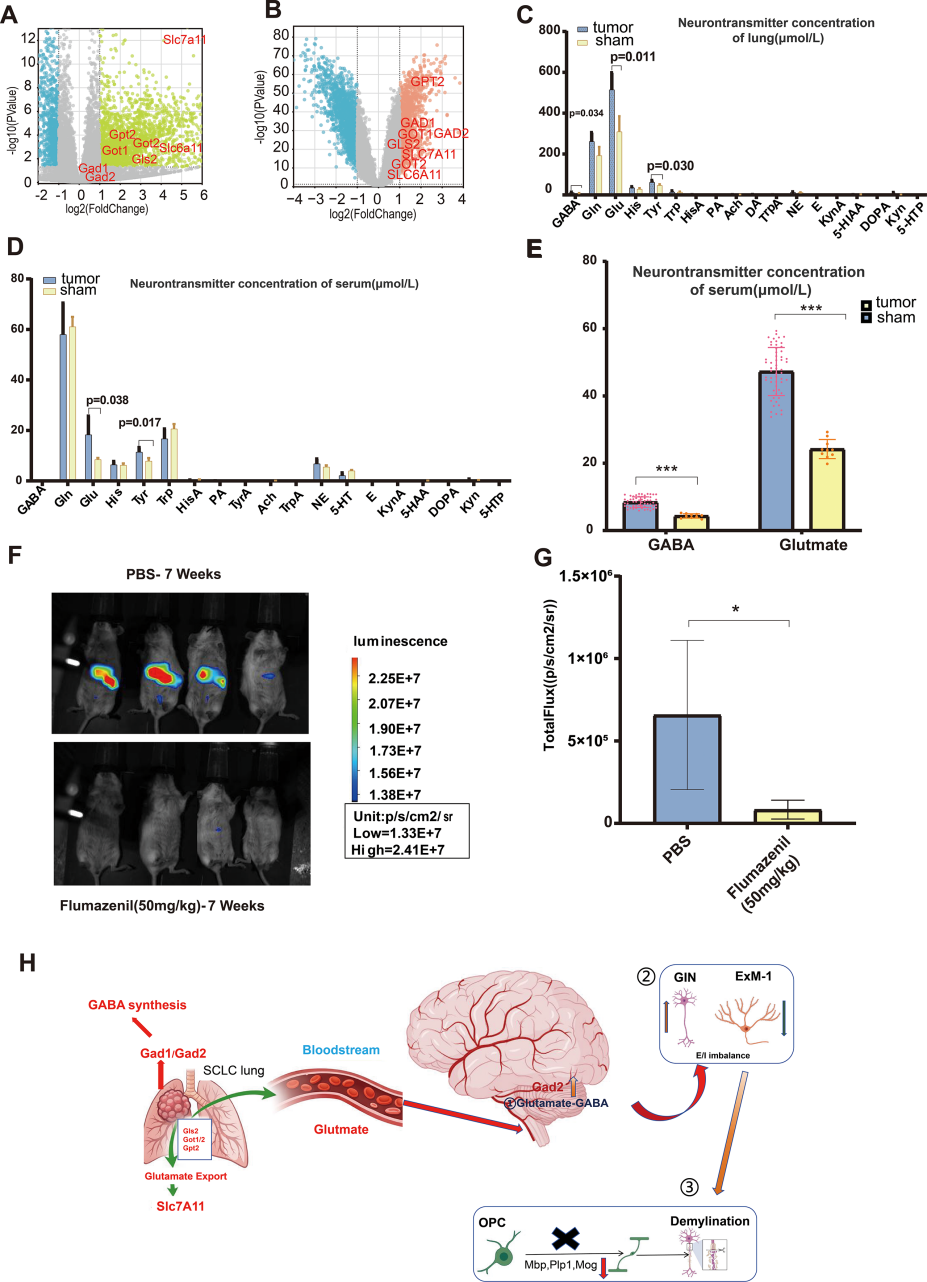
SCLC may drive remodeling of the brain microenvironment via a circulating glutamate signaling axis. Volcano plot of differentially expressed genes between lung tumors from tumor-bearing mice (tumor) and sham-treated controls(sham), highlighting key genes involved in glutamate and GABA metabolism and transport, including *Gls2, Got1, Got2, Gpt2, Slc7a11, Gad1, Gad2*, and *Slc6a11*, *Gad1* and *Gad2* transcripts showed an upward trend in tumor tissues (log_2_FC = 0.46 and 0.71, respectively), but this difference did not reach the pre-specified threshold of statistical significance (*Gad1*: adjusted P = 0.23; *Gad2*: adjusted P = 0.79) **(A)**. Volcano plots highlighting significant alterations in genes related to glutamate and GABA metabolism and transport following batch-corrected integration of the HRA003419 and GSE60052 cohorts **(B)**. Quantitative metabolomics of neurotransmitters in lung tissues from an SCLC mouse model, revealing elevated levels of both glutamate and GABA **(C)**. Quantitative metabolomics of neurotransmitters in the serum of tumor-bearing mice. GABA was below the detection limit (ND) in all samples. Data represent biological replicates. Statistical significance was determined by two-tailed Student’s t-test (*P < 0.05, **P < 0.01, and ***P < 0.001) **(D)**. Quantification of GABA and glutamate levels by ELISA in the plasma of SCLC patients, showing significant elevation of both neurotransmitters, with glutamate concentrations far exceeding those of GABA, GABA was quantifiable in patient plasma by ELISA, in contrast to the mouse serum LC−MS/MS results; this difference may reflect variation in assay sensitivity or specificity across platforms. **(E)**. Experimental design for therapeutic intervention: RPM mice were induced to develop SCLC via intratracheal adenovirus administration and treated with a GABA_A_ receptor antagonist (Flumazenil; 50 mg/kg) or PBS vehicle control **(F)**. Statistical analysis of *in vivo* fluorescence imaging intensity. Asterisks indicate *P* < 0.05, and ‘ns’ indicates *P* ≥ 0.05 (Wilcoxon rank-sum test) **(G)**. Schematic model illustrating the mechanisms by which the SCLC-derived circulating glutamate axis influences brain microenvironment remodeling **(H)**.

To investigate the potential source of this axis, we first analyzed lung tumors from the RPM mouse model using transcriptomic and targeted metabolomic profiling (**Supplementary Figure 7A**). Pathway analysis revealed significant enrichment of glutamate and GABA biosynthesis, indicating pronounced metabolic rewiring within the tumor microenvironment. These findings suggest that primary tumors may generate circulating metabolites capable of influencing distant organs. Interestingly, *Gad1* and *Gad2* transcripts showed an upward trend in tumor tissues (log_2_FC = 0.46 and 0.71, respectively), but this difference did not reach the pre-specified threshold of statistical significance (*Gad1*: adjusted P = 0.23; *Gad2*: adjusted P = 0.79; **Figure 5A**), which may partly reflect sampling heterogeneity and limited sample size. In contrast, genes related to glutamate synthesis—*Gls2*, *Got1*, *Got2*, and *Gpt2*—as well as the transporter gene *Slc7a11*, were significantly upregulated (adjusted P < 0.05, |log_2_FC| > 1) (**Figure 5A**). KEGG pathway enrichment further highlighted activation of neuroactive ligand signaling, neuroactive ligand-receptor interaction, GABAergic synapse, and Glutamatergic synapse (**Supplementary Figure 7B**), consistent with enhanced neurotransmitter-related signaling within tumors. Metabolomic measurements confirmed accumulation of both glutamate and GABA in tumor tissue, with glutamate present at substantially higher levels (**Figure 5C****;** **Supplementary Table 1**). In circulation, however, glutamate remained significantly elevated whereas GABA was below the detection limit of the LC−MS/MS assay in all serum samples (**Figure 5D****;** **Supplementary Table 2**), indicating that circulating GABA, if present, is at concentrations too low to be detected by this method.

We next assessed whether similar patterns were observed in human SCLC. Integrated analysis of public transcriptomic datasets (GSE60059 and the HRA003419) was performed following batch correction using SVA (45) and ComBat normalization, with principal component analysis confirming effective harmonization. **Supplementary Figures 7C, D**. Differential expression analysis demonstrated general upregulation of genes involved in glutamate and GABA synthesis, including GAD1, GAD2, GLS2, GOT1, and GPT2 (P<0.05, log_2_FC>1), in tumor samples. In contrast, expression of transporters mediating extracellular GABA export, such as *SLC6A11*, was relatively low (**Figure 5B**), suggesting that tumor-derived GABA may primarily act locally, whereas glutamate is more readily released into circulation. Consistent with this interpretation, ELISA measurement in patient plasma showed increased levels of both neurotransmitters, with a substantially greater elevation in glutamate (**Figure 5E**). Together, these findings indicate distinct systemic behaviors of glutamate and GABA during SCLC progression, with glutamate more prominently represented in circulation and GABA largely confined to the tumor microenvironment (46).

### Targeting GABAergic signaling suppresses SCLC tumor growth

Previous studies have demonstrated that SCLC cells receive both glutamatergic and GABAergic synaptic inputs that promote tumor cell proliferation through distinct mechanisms (22). Because SCLC cells maintain elevated intracellular chloride levels, activation of GABA_A_ receptors can induce depolarization and increased cellular excitability. Pharmacological modulation of glutamatergic signaling, including treatment with the GRM8 agonist (DCPG) or the glutamate release inhibitor (riluzole), has been reported to significantly suppress tumor growth and improve survival in preclinical models, with riluzole showing synergistic effects with chemotherapy (etoposide + cisplatin) (22). However, the basic biology of GABAergic inputs in SCLC is recognized, its therapeutic exploitation has received disproportionately less attention. To address this, we treated RPM mice(n=4vs4) with a GABA_A_ receptor antagonist administered intraperitoneally (50 mg/kg every three days). Tumor growth was significantly reduced by seven weeks following tumor induction (**Figures 5F, G**), supporting a contributory role for GABAergic signaling in tumor progression. Notably, SCLC cells themselves express GABA_A_ receptors (22), and flumazenil exhibits limited penetration of the blood–brain barrier. Therefore, its tumor-suppressive effect most likely arises from direct inhibition of autocrine/paracrine GABA signaling within the tumor microenvironment, rather than from modulation of central GABAergic circuits. This result represents an independent pharmacological observation and does not serve as direct evidence for the lung–brain axis.

Taken together, our multi-omics data support a model in which glutamate and GABA contribute to SCLC biology through distinct spatial mechanisms. Glutamate appears to act as a circulating signal capable of influencing distant brain physiology, whereas GABA primarily functions within the tumor microenvironment. Whether circulating glutamate directly promotes ectopic *Gad2*-associated transcriptional changes and altered inhibitory signaling programs in the brain remains to be determined. Our data establish a correlative association between tumor metabolism and brain remodeling, while future interventional studies will be required to test this causal model (**Figure 5I**).

## Discussion

In this study, we used whole-brain single-nucleus RNA sequencing to systematically define the transcriptional changes of the brain in a mouse model of SCLC. This approach enabled unbiased profiling of major neural and non-neural cell populations and revealed widespread, cell-type-specific transcriptional remodeling associated with tumor development. Among the most prominent changes, neuronal populations—particularly GABAergic inhibitory neurons-exhibited marked upregulation of *Gad2*, accompanied by a global shift in excitatory–inhibitory related transcriptional programs. In contrast, non-neuronal lineages, most notably the oligodendrocyte lineage, displayed coordinated downregulation of core myelination genes, including *Mbp*, *Plp1*, and *Mog*. These divergent patterns suggest that SCLC elicits distinct transcriptional responses in neuronal versus glial compartments, with enhanced GABAergic potential in neurons and impaired myelination programs in oligodendroglia. These findings collectively highlight multiple molecular entry points through which SCLC may perturb brain homeostasis.

By integrating transcriptomic and metabolomic data obtained from both clinical samples and mouse models, we propose a model in which tumor-associated elevations in circulating glutamate may influence brain physiology. Elevated glutamate may promote increased *Gad2* expression and enhanced conversion of glutamate to GABA within the brain, contributing to excitation–inhibition imbalance and disruption of neuron–glia communication pathways, including NRG–ERBB4 signaling. These alterations are associated with transcriptional signatures of impaired OPC maturation and reduced myelination, providing a putative mechanistic link between tumor metabolism and structural brain changes that requires future experimental validation.

To explore the systemic cues that might drive these vascular changes, we integrated proteomic data from SCLC patient sera with secretome profiles from a panel of SCLC cell lines. This intersection identified VEGFA as a prominently elevated circulating factor, consistent with its established role as a mediator of BBB permeability. The high expression of its cognate receptor Flt1 in brain endothelial cells further supports the notion that tumor-derived VEGFA may directly engage the cerebral endothelium. While our findings do not establish causality, they provide a plausible molecular link between peripheral tumor metabolism and the transcriptional reprogramming observed in the brain vasculature.

Therapeutic potential of targeting GABAergic signaling in SCLC: Beyond the lung–brain metabolic axis proposed above, our flumazenil experiment reveals a distinct pharmacological finding. SCLC cells are known to express functional GABA_A_ receptors, and flumazenil has limited penetration across the blood–brain barrier. Therefore, the tumor suppression we observed most likely reflects direct blockade of autocrine/paracrine GABA signaling within the tumor microenvironment, rather than modulation of central GABAergic circuits. This suggests that tumor-intrinsic GABAergic signaling contributes to SCLC proliferation and that pharmacological targeting of this peripheral GABAergic loop may represent an independent therapeutic strategy for SCLC. Notably, this finding is separate from the hypothesized lung–brain communication pathway and should be evaluated on its own merits in future preclinical studies, including testing of GABA_A_ antagonists with optimized pharmacokinetic profiles.

Several limitations should be addressed in future work. First, our analysis focuses on whole-brain changes and did not resolve region-specific responses. Given the functional heterogeneity of brain regions, future studies incorporating spatial transcriptomics or region-targeted profiling will be important to determine whether specific circuits are preferentially affected. Nevertheless, we sought to infer potential regional vulnerability from the functional identity of the dysregulated genes. The pronounced downregulation of core myelination genes (*Mbp, Plp1, Mog*) in the oligodendrocyte lineage suggests that myelin loss may preferentially affect cognition-relevant regions such as the hippocampus and cerebral cortex, where efficient myelination is critically required. Second, a key limitation of this study is that the proposed causal chain—from tumor-derived circulating glutamate to *Gad2* upregulation, excitation–inhibition imbalance, and impaired OPC maturation in the brain—has not been directly established. The current evidence is correlative. Future *in vivo* rescue experiments, such as pharmacological inhibition of glutamate synthesis (e.g., with L-methionine sulfoximine) or transport (e.g., with erastin) in tumor-bearing mice, will be necessary to test whether reducing circulating glutamate can block or reverse these brain transcriptional changes. Third, the sample size in our single-nucleus RNA sequencing experiment was limited, which may reduce statistical power and obscure inter-individual variability. Fourth, we cannot fully exclude the possibility that some of the observed brain transcriptomic changes are secondary to non-specific systemic effects of tumor burden, such as systemic inflammation, cytokine release (e.g., IL-6, TNF-α), cachexia-associated metabolic stress, or anemia-related hypoxia. These factors could independently influence brain gene expression. Future studies using non-cachectic tumor models or controlling for circulating inflammatory mediators will be required to disentangle tumor-specific signals from general sickness responses. Fifth, all animal experiments in this study were performed exclusively in female mice. Given known sex differences in SCLC incidence and progression, the generalizability of these findings to males remains to be tested. Future studies using pharmacological blockade of glutamate signaling or cell-type-specific genetic approaches will be required to determine whether modulation of glutamate alone is sufficient to induce or reverse *Gad2* expression, excitation–inhibition balance, and oligodendrocyte differentiation. Similarly, the functional contribution of *Gad2* in distinct brain cell populations, including endothelial cells, warrants further investigation using conditional loss-of-function models. Additionally, the detection of GABA in patient plasma by ELISA (**Figure 5E**) but not in mouse serum by LC−MS/MS (**Supplementary Table 2**) may reflect differences in assay sensitivity, matrix effects, or antibody cross-reactivity between the two platforms; direct cross-platform validation was not performed in this study.

From a translational perspective, our findings reveal a significant association between SCLC and both brain transcriptional remodeling and elevated circulating glutamate, supporting the existence of a lung–brain metabolic axis. However, the direct causal relationships within this axis remain to be established. Given that SCLC cells depend on glutamatergic and GABAergic inputs for proliferation, interventions targeting tumor-associated neurotransmitter signaling might simultaneously impact tumor growth and potentially distal neural dysfunction, though this possibility requires further mechanistic validation. Notably, the tumor-suppressive effect of flumazenil observed in this study likely reflects direct pharmacological blockade of GABA_A_ receptors on tumor cells, representing an independent therapeutic strategy rather than evidence for the lung–brain axis. Future studies may explore combined strategies that target tumor-derived metabolic factors alongside pathways that maintain vascular and glial homeostasis, with the goal of mitigating tumor-associated neurological impairment and preserving brain integrity.

## Data Availability

The datasets presented in this study can be found in online repositories. The names of the repository/repositories and accession number(s) can be found in the article/**Supplementary Material**.
