# Supplementary material for: Critical changes in whole-brain gene networks in response to small-cell lung cancer as revealed by single-nucleus RNA sequencing
Source: Front Immunol. 2026 Jun 2;17:1860628. doi: 10.3389/fimmu.2026.1860628 (PMC13269077; doi:10.3389/fimmu.2026.1860628)
Supplement: Supplementary file 1 [file DataSheet1.docx]

**
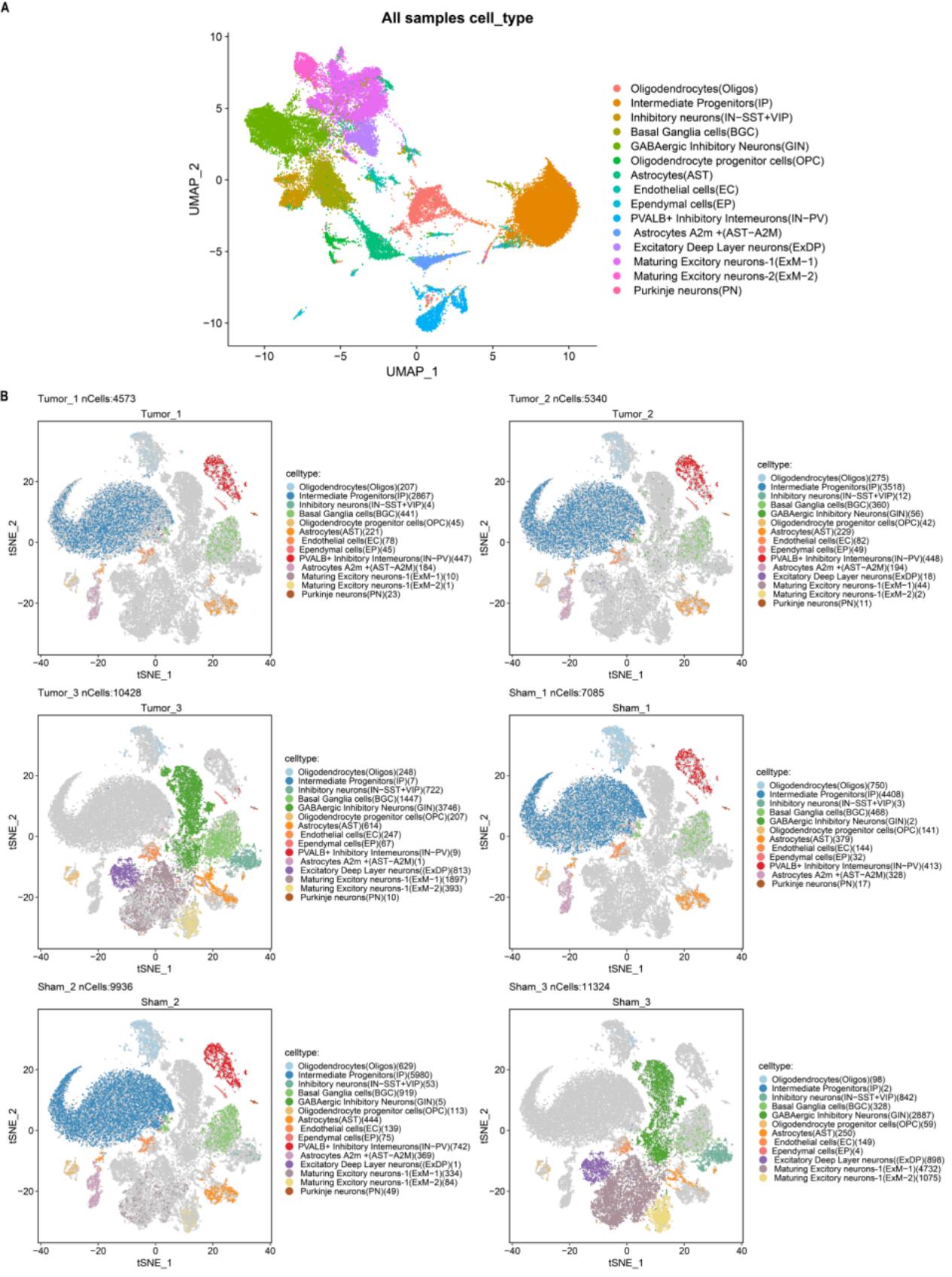
**

**Supplementary Figure 1. Visualization of whole-brain single-nucleus transcriptomes from tumor-bearing and control mice.**

.UMAP projections of all 48,686 nuclei from all samples (three tumor-bearing mice and three sham-treated controls)(A).t-SNE projections of all 48,686 nuclei were split by individual sample (three tumor-bearing mice and three sham-treated controls). This per-sample view illustrates the overall consistency of cell-type representation across biological replicates, while also highlighting sample-to-sample variation in relative cell-type abundance(B).

**
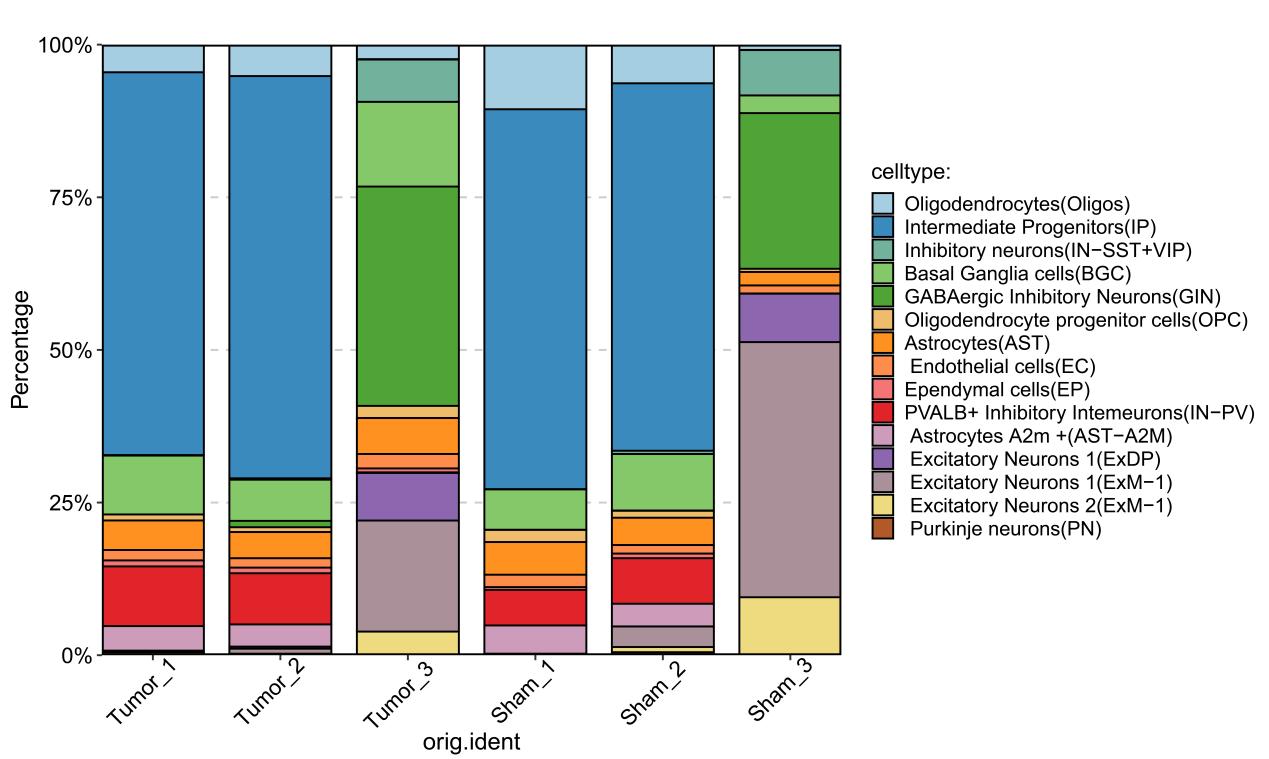
**

**Supplementary Figure 2.Relative cell type proportions (percentage) shown for each of the six individual mice.**

Stacked bar plots showing the relative proportions of major cell types in whole-brain single-nucleus transcriptomes from individual mice. Samples are grouped as sham-treated controls (Sham1–Sham3) and tumor-bearing mice (Tumor1–Tumor3). While cell-type proportions are largely consistent across biological replicates, minor deviations in the representation of specific neuronal subpopulations are observed in samples Cre3 and WT3, likely attributable to the high myelin content of adult brain tissue and the preferential loss of fragile cell subsets during nuclear isolation.

**
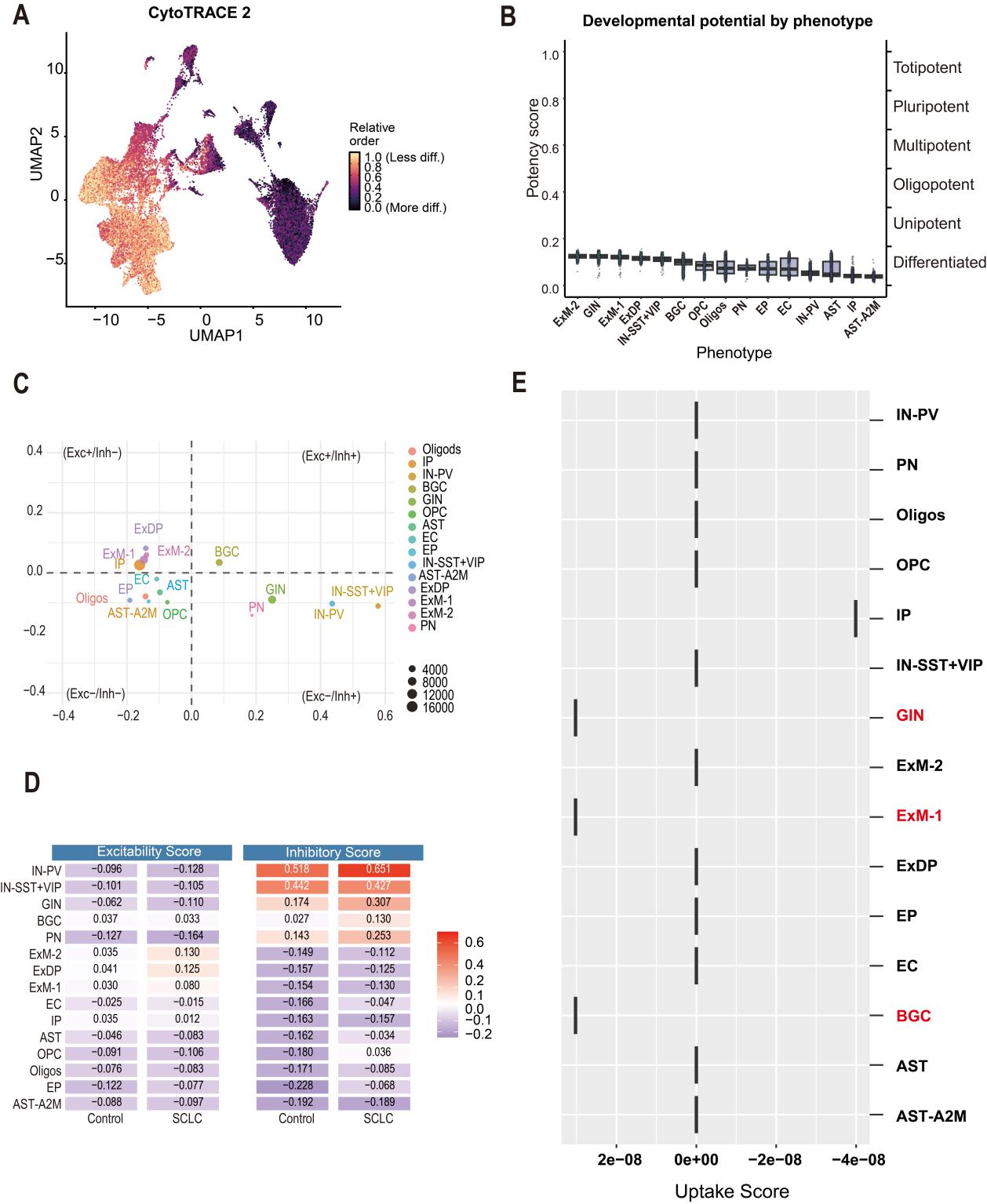
Supplementary Figure 3. Characterization of cellular differentiation potential, excitatory-inhibitory balance, and metabolic activity across brain cell subpopulations.**

Cellular differentiation potential was predicted using CytoTRACE, identifying ExM-1, GINs, and ExM-2 as the most prominent populations (**A-B**).Cell subpopulations were classified based on excitatory and inhibitory module scores. These scores were calculated for all cells using excitatory neuron markers (*Slc17a7*, *Slc17a6, Slc17a8*) and inhibitory neuron markers (*Gad1*, *Gad2*, *Slc32a1*) via the AddModuleScore function (**C**).Comparative analysis revealed changes in excitatory and inhibitory module scores across different cell subpopulations between the tumor and sham groups (**D**).Metabolic flux inference using the METAFlux algorithm indicated upregulation of glucose uptake-related pathways in BGC, ExM-1, and GIN populations (**E**).


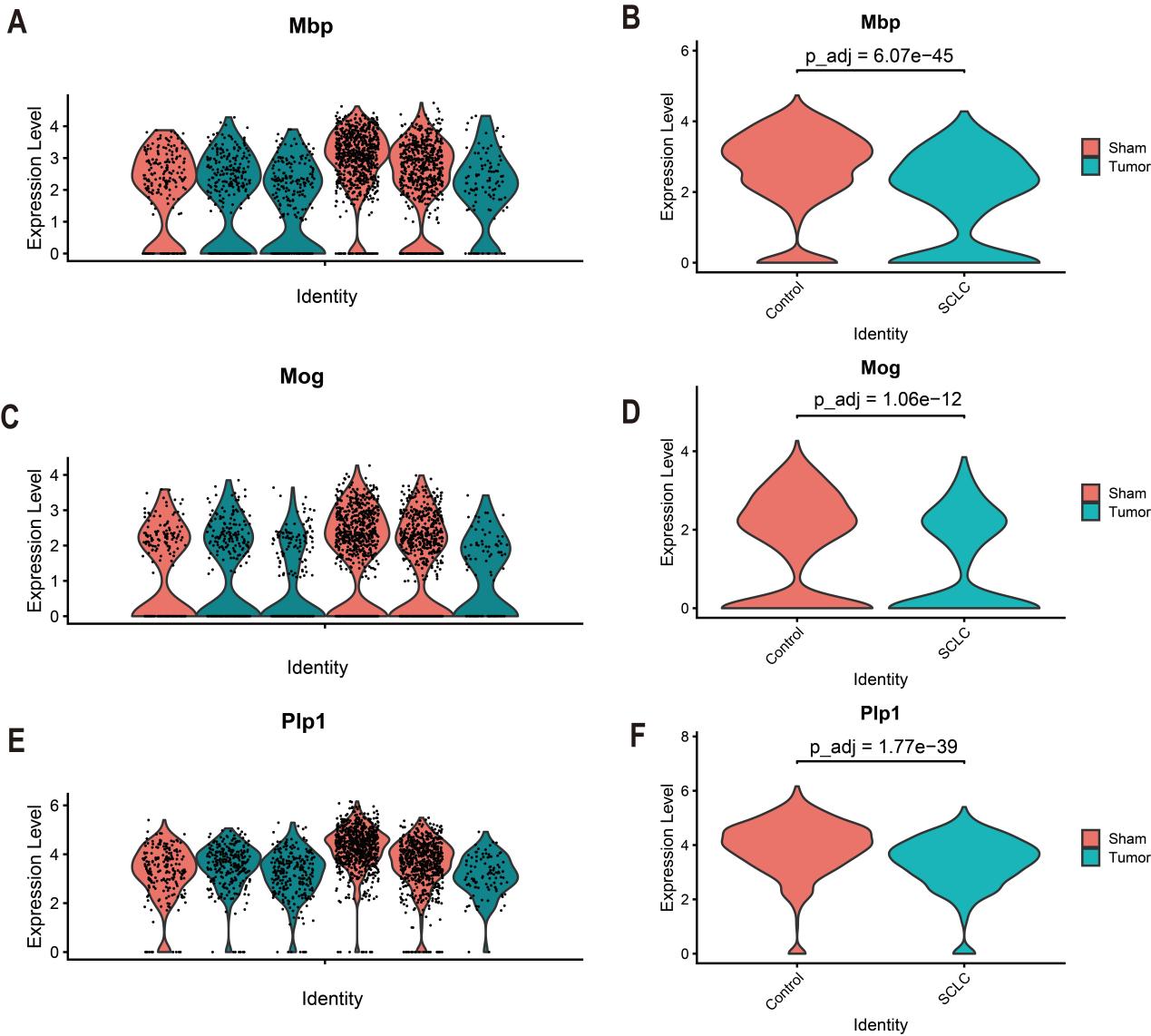


**Supplementary Figure 4. Violin plots showing expression of Mbp, Mog, and Plp1 in oligodendrocytes.**

Expression distribution of *Mbp* across the six individual mice (**A**) .Comparison of *Mbp* expression between sham-treated control and tumor-bearing groups, with statistical significance indicated(**B**) . Expression distribution of *Mog* across the six individual mice(**C**) . Comparison of *Mog* expression between sham-treated control and tumor-bearing groups, with statistical significance indicated(**D**) . Expression distribution of *Plp1* across the six individual mice(**E**) . Comparison of *Plp1* expression between sham-treated control and tumor-bearing groups, with statistical significance indicated(**F**) .Statistical significance was assessed using the Wilcoxon rank-sum test, and adjusted P values (p_adj) are shown.


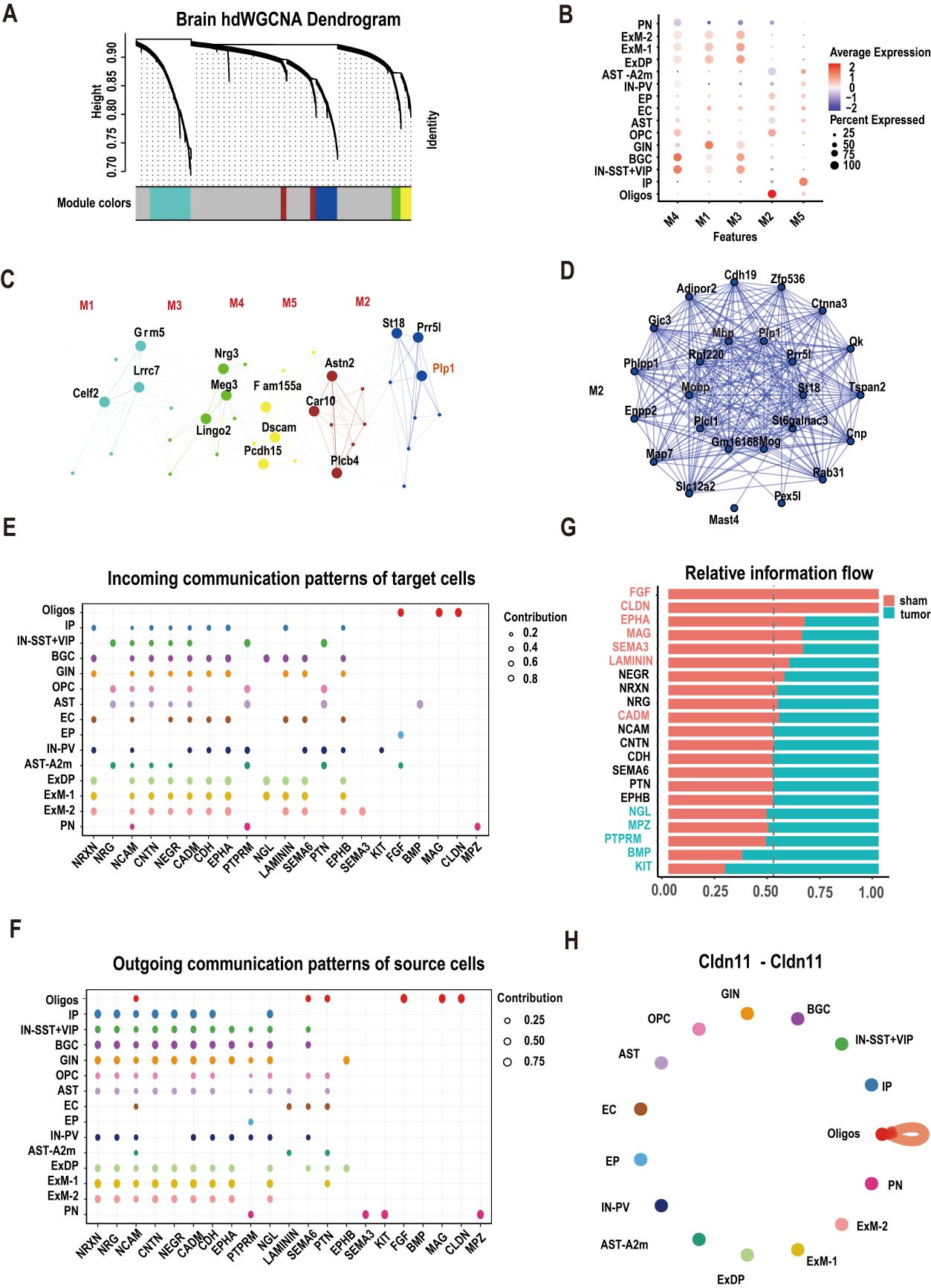


**Supplementary Figure 5. From genomic modules to altered cellular crosstalk: SCLC remodels brain glial networks and impairs myelination-related signaling.**

WGCNA module–trait relationship analysis. Heatmap showing five gene co-expression modules and their correlations with the SCLC condition(**A**) .Cell type-specific module expression. Dot plot illustrating the average expression of each WGCNA module across major brain cell types, highlighting the enrichment of Module 2 in oligodendrocytes and oligodendrocyte precursor cells (OPCs)(**B**).Representative genes from WGCNA modules. Key genes from different modules are shown(**C**).Co-expression network of Module 2. Network graph depicting gene nodes and their co-expression connections. *Mbp* and *Plp1* are highlighted as hub genes by larger node sizes and distinct colors(**D**).Clustering analysis of signaling output and input patterns. CellChat analysis reveals distinct communication patterns between cell populations(**E-F**).Differential cell-cell communication in SCLC. Comparative analysis between tumor and sham groups identifies significantly downregulated signaling pathways, including FGF, claudin (CLDN), ephrin (EPHA), and myelin-associated glycoprotein (MAG). Among these, FGF, CLDN, and MAG are associated with Pattern 2 signaling and oligodendrocyte-related communication. CLDN signaling plays a critical role in both myelination and blood-brain barrier integrity(**G**) .Visualization of the CLDN signaling pathway. Schematic representation of claudin-mediated signaling interactions altered in the SCLC microenvironment(**H**).

**
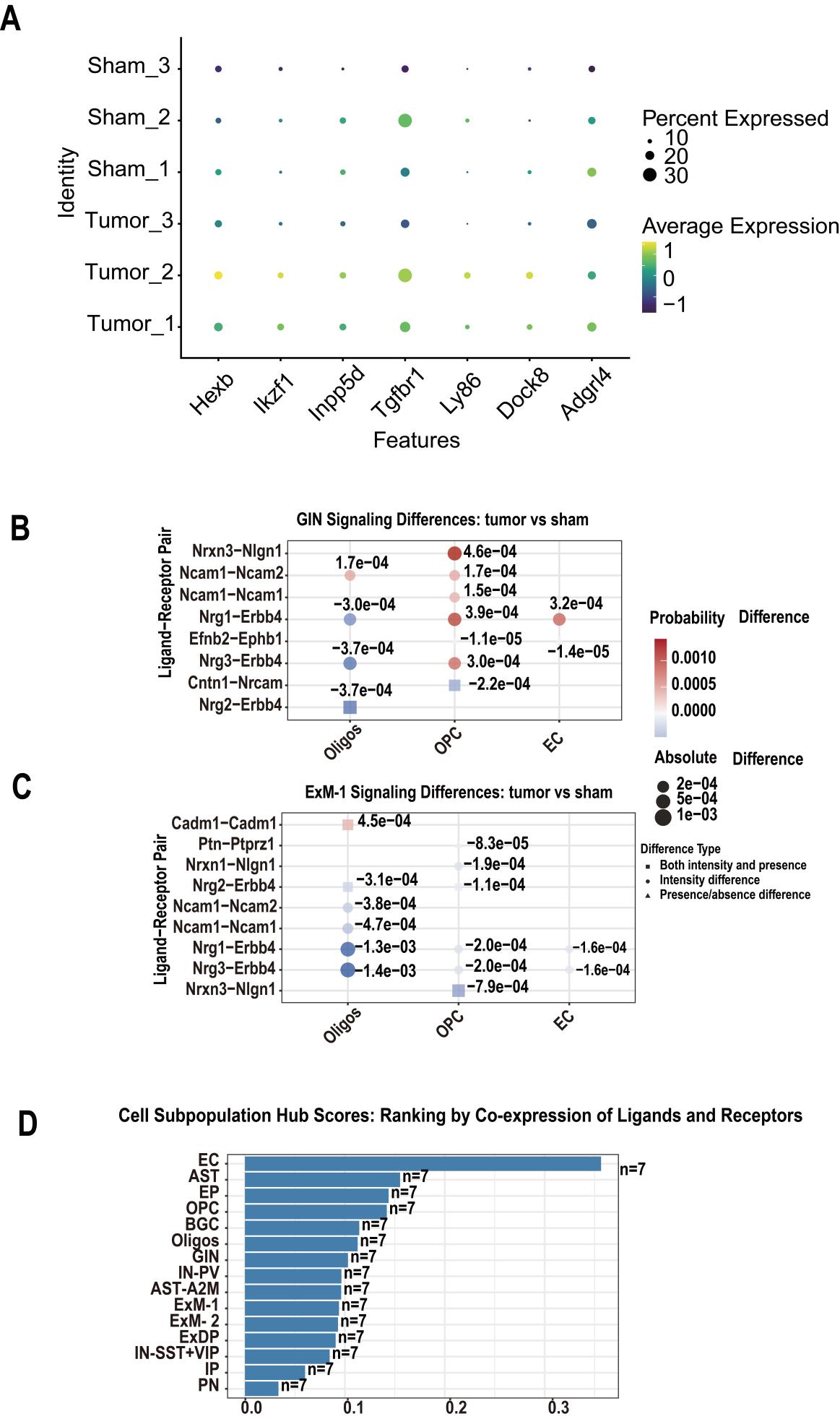
**

**Supplementary Figure 6:Inflammatory endothelial states and altered neuron-glia-vascular communication in SCLC**.

Dot plot depicting the expression patterns of the top five marker genes for the MP1 endothelial metaprogram(**A**).Differences in CellChat-inferred interactions between GABAergic inhibitory neurons and OPCs, oligodendrocytes, and endothelial cells in tumor-bearing mice(tumor) versus sham-treated controls(sham); red indicates upregulated interactions, and blue indicates downregulated interactions(**B**). Differences in CellChat-inferred interactions between ExM-1 and OPCs, oligodendrocytes, and endothelial cells in tumor-bearing mice(tumor) versus sham-treated controls(sham); red indicates upregulated interactions, and blue indicates downregulated interactions(**C**) .Bar plot showing average scores for cells co-expressing both ligands and receptors across subpopulations, with endothelial cells showing the highest scores, followed by astrocytes (AST) and ependymal cells (EP), consistent with the composition of blood-brain barrier-related cells(**D**) .


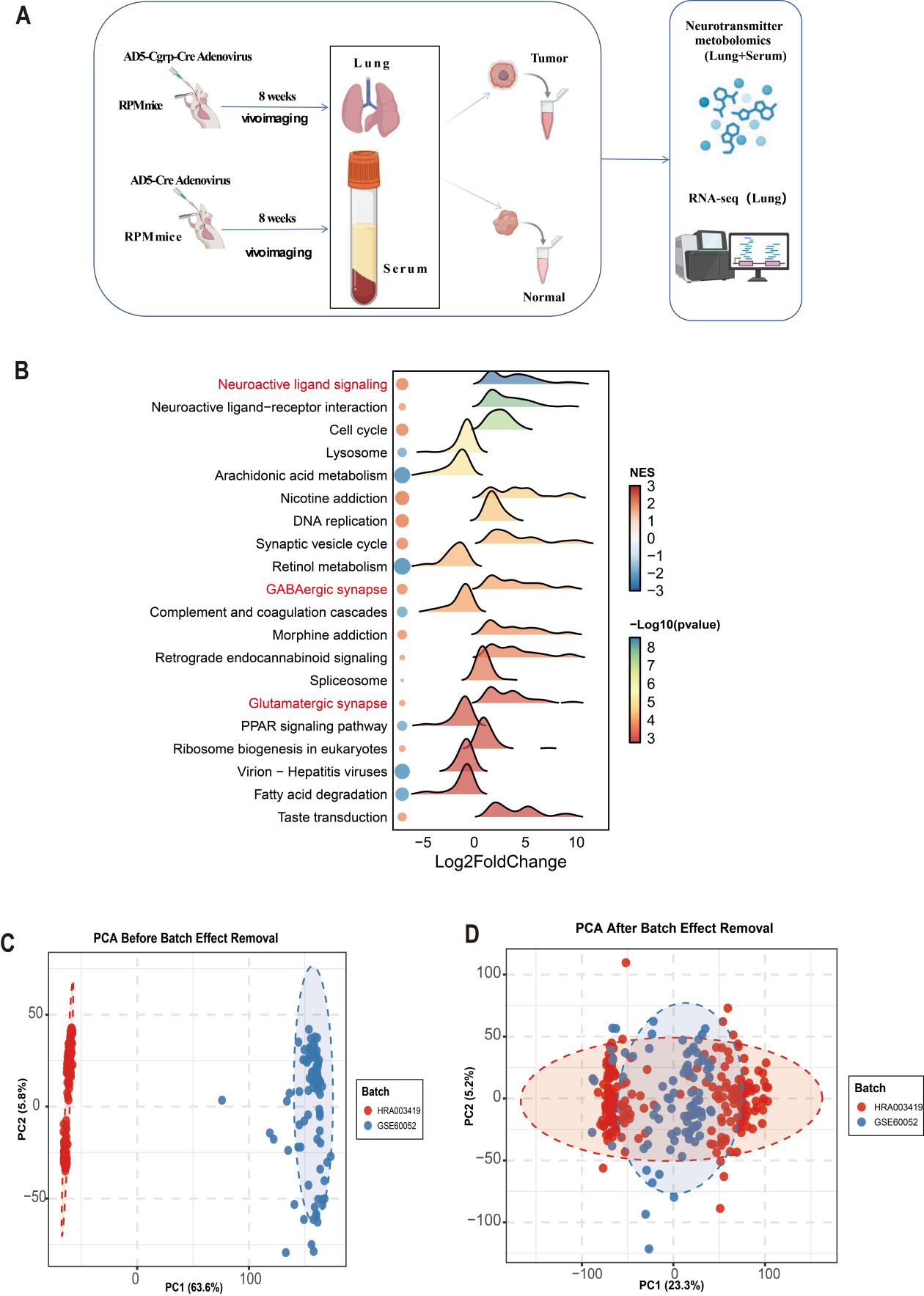


**Supplementary Figure 7:SCLC Datasets and Validation in Mouse Models Reveals Enrichment of GABAergic Synapse, Glutamatergic Synapse, and Neurotransmitter-Related Signaling Pathways in Lung Tissues**.

Schematic diagram of sample collection from RPM model mice for targeted neurotransmitter metabolomics analysis (4 vs 4) and transcriptomics analysis (5 vs 5) (**A**). GSEA-KEGG analysis shows the top 20 pathways enriched in the transcriptomics data(**B**)**.** PCA scatter plot shows distinct separation between TSU-SCLC and GSE60052 datasets before batch correction, indicating batch effects**(C)**.PCA scatter plot after batch correction shows the integration of TSU-SCLC and GSE60052datasets, indicating reduced batch effects**(D)** .

**Supplementary Tables**

**Supplementary Table1:Neurotransmitter levels in lung tissues from tumor-bearing mice(tumor) versus sham-treated controls(sham)**

| sample | tumor-1 | tumor-2 | tumor-3 | tumor-4 | sham-1 | sham-2 | sham-3 | sham-4 |
| --- | --- | --- | --- | --- | --- | --- | --- | --- |
| GABA | 8.307 | 10.835 | 11.796 | 13.782 | 8.511 | 7.810 | 6.077 | 1.955 |
| Gln | 258.552 | 243.323 | 322.613 | 219.663 | 177.851 | 255.323 | 172.473 | 163.405 |
| Glu | 526.425 | 455.848 | 627.749 | 448.791 | 289.463 | 421.291 | 270.531 | 255.712 |
| His | 30.247 | 40.019 | 38.297 | 29.300 | 28.707 | 34.956 | 24.856 | 22.229 |
| Tyr | 53.323 | 74.765 | 59.605 | 58.664 | 44.539 | 53.559 | 47.058 | 40.320 |
| Trp | 8.599 | 20.867 | 9.322 | 16.732 | 12.105 | 16.796 | 14.732 | 12.115 |
| HisA | 0.508 | 0.595 | 1.021 | 0.531 | 0.660 | 0.702 | 0.604 | 0.386 |
| PA | 0.226 | 0.244 | 0.307 | 0.263 | 0.179 | 0.276 | 0.147 | 0.159 |
| TyrA | ND | ND | ND | ND | ND | ND | ND | ND |
| Ach | 0.379 | 0.709 | 0.533 | 0.802 | 0.346 | 0.503 | 0.244 | 0.265 |
| DA | 0.002 | 2.367 | 0.003 | 0.003 | 0.001 | 0.003 | 0.001 | 0.001 |
| TrpA | 0.050 | 0.967 | 0.057 | 0.008 | 0.009 | 0.006 | 0.012 | 0.091 |
| NE | 13.831 | 11.236 | 15.266 | 8.835 | 12.036 | 11.815 | 9.999 | 12.263 |
| 5-HT | ND | ND | ND | ND | ND | ND | ND | ND |
| E | 0.116 | 0.127 | 0.202 | 0.094 | 0.114 | 0.183 | 0.065 | 0.091 |
| KynA | 0.020 | 0.024 | 0.026 | 0.020 | 0.011 | 0.011 | 0.005 | 0.007 |
| 5-HIAA | 0.313 | 0.809 | 0.428 | 0.662 | 0.820 | 1.922 | 1.615 | 1.002 |
| DOPA | 0.144 | 0.201 | 0.244 | 0.170 | 0.133 | 0.192 | 0.073 | 0.105 |
| XA | ND | ND | ND | ND | ND | ND | ND | ND |
| Kyn | 10.638 | 0.594 | 11.817 | 0.613 | 0.212 | 0.923 | 0.629 | 0.207 |
| VMA | ND | ND | ND | ND | ND | ND | ND | ND |
| 5-HTP | 0.020 | 0.012 | 0.008 | 0.007 | 0.007 | 0.007 | 0.003 | 0.005 |
| MT | ND | ND | ND | ND | ND | ND | ND | ND |

*ND indicates not detected

**Supplementary Table2:Serum neurotransmitter levels in tumor-bearing mice(tumor) versus sham-treated controls(sham)**

| sample | tumor-1 | tumor-2 | tumor-3 | tumor-4 | sham-1 | sham-2 | sham-3 | sham-4 |
| --- | --- | --- | --- | --- | --- | --- | --- | --- |
| GABA | ND | ND | ND | ND | ND | ND | ND | ND |
| Gln | 70.550 | 66.812 | 47.013 | 64.206 | 55.408 | 61.680 | 62.944 | 47.492 |
| Glu | 26.039 | 23.076 | 11.364 | 8.637 | 7.787 | 9.303 | 8.128 | 12.593 |
| His | 7.654 | 7.403 | 5.167 | 5.618 | 5.798 | 7.243 | 6.162 | 5.429 |
| Tyr | 9.727 | 10.050 | 12.509 | 7.124 | 7.206 | 9.688 | 7.452 | 13.356 |
| Trp | 13.080 | 13.669 | 19.325 | 20.993 | 21.339 | 22.269 | 17.859 | 20.685 |
| HisA | 0.327 | 0.347 | 0.319 | 0.391 | 0.629 | 0.531 | 0.445 | 0.345 |
| PA | 0.017 | 0.020 | 0.039 | 0.041 | 0.043 | 0.045 | 0.039 | 0.037 |
| TyrA | ND | ND | ND | ND | ND | ND | ND | ND |
| Ach | 0.060 | 0.053 | 0.050 | 0.049 | 0.039 | 0.035 | 0.036 | 0.054 |
| DA | ND | ND | ND | ND | ND | ND | ND | ND |
| TrpA | 0.002 | 0.001 | 0.004 | 0.002 | 0.001 | 0.001 | 0.001 | 0.001 |
| NE | 8.909 | 7.886 | 5.281 | 6.483 | 5.426 | 5.194 | 4.990 | 5.123 |
| 5-HT | 1.052 | 1.397 | 2.972 | 3.711 | 4.058 | 4.444 | 3.796 | 3.137 |
| E | 0.021 | 0.022 | 0.006 | 0.012 | 0.020 | 0.018 | 0.010 | 0.004 |
| KynA | 0.018 | 0.020 | 0.032 | 0.017 | 0.010 | 0.014 | 0.017 | 0.025 |
| 5-HIAA | 0.116 | 0.105 | 0.105 | 0.102 | 0.101 | 0.068 | 0.095 | 0.109 |
| DOPA | 0.035 | 0.033 | 0.034 | 0.033 | 0.028 | 0.027 | 0.034 | 0.031 |
| XA | ND | ND | ND | ND | ND | ND | ND | ND |
| Kyn | 0.757 | 0.659 | 0.201 | 0.345 | 0.185 | 0.207 | 0.224 | 0.206 |
| VMA | ND | ND | ND | ND | ND | ND | ND | ND |
| 5-HTP | 0.001 | 0.001 | 0.001 | 0.002 | 0.002 | 0.002 | 0.001 | 0.002 |
| MT | ND | ND | ND | ND | ND | ND | ND | ND |

*ND indicates not detected
